# Effect of bonded lingual spurs with low trans palatal arch on developing anterior open bite (a randomized controlled trial)

**DOI:** 10.1186/s12903-026-07717-9

**Published:** 2026-02-13

**Authors:** Moataz Nasser, Tarek Nasreldin Yousry, Hanan Amin Ismail

**Affiliations:** Department of Orthodontics, Faculty of Dentistry, Alexandria University, Alexandria, Egypt

**Keywords:** Anterior open bite, Early treatment, Low transpalatal arch, Tongue spurs

## Abstract

**Background:**

Anterior Open Bite (AOB) is a complex malocclusion with esthetic, functional, and psychosocial implications, highlighting the need for early diagnosis and interceptive treatment.

**Objective:**

To compare the effectiveness of bonded Tongue Spurs combined with a Low Trans Palatal Arch (TS/LTPA) versus bonded Tongue Spurs (TS) alone in managing AOB associated with tongue habit during the mixed dentition stage.

**Materials and methods:**

Fifty children were assessed for eligibility; 32 were randomized, and 30 completed the 6-month follow-up and were included in the final analysis. They aged 7 to 11 years (10 boys, 20 girls) in the mixed dentition phase, having a dentoskeletal AOB of at least 1 mm, an Angle Class I molar relationship, and CMI stages 2 and 3. TS/LTPA group comprised 16 patients (7 males and 9 females) exhibiting bonded lingual tongue spurs and a low transpalatal arch. TS group included 14 patients (3 males and 11 females) who had bondable lingual tongue spurs only. The mean age was 8.73 ± 1.00 years in the TS/LTPA group and 9.03 ± 0.68 years in the TS group. Digital lateral cephalograms and models were obtained prior to and six months post-treatment.

**Results:**

Both groups (TS/LTPA and TS) showed improvement in overbite closure, with a median change of + 2.97 mm (IQR 1.16 to 5.00; *p* < .001) in the TS/LTPA group and + 3.67 mm (IQR 2.00 to 5.00; *p* = .001) in the TS group, respectively. However, the difference between groups was not statistically significant. A statistically significant vertical molar control was observed in the TS/LTPA group (+ 0.40 mm, IQR 0.30 to 0.65; *p* = .001) compared to the TS group (+ 0.90 mm, IQR 0.70 to 1.00; *p* = .001), with significant differences between both groups.

**Conclusion:**

Both TS/LTPA and TS groups showed a reduction in overbite, along with incisor extrusion and retroclination. The TS/LTPA group provided significantly better vertical molar control than the TS group; however, the difference in AOB closure between the groups was not statistically significant.

**Trial registration:**

This single-center randomized controlled trial received ethical approval from Alexandria University’s Research Ethics Committee, Alexandria, Egypt (IORG0008839, No. 0313 − 10/2021). This trial was retrospectively registered in the Pan African Clinical Trial Registry (PACTR202403582913859) on 14 March 2024.

**Supplementary Information:**

The online version contains supplementary material available at 10.1186/s12903-026-07717-9.

## Background

Anterior Open Bite (AOB) is characterized by the lack of vertical overlap between the upper and lower anterior teeth while the posterior teeth are in occlusion [1, 2]. The prevalence of open bite fluctuates with age, with a notable incidence of 17.7% during the mixed dentition phase [3, 4].

Developing anterior open bite is strongly associated with an aberrant swallowing pattern characterized by anterior tongue rest posture and reduced tongue–palate contact, which preserve dentoalveolar imbalance. Speech articulation is frequently affected, with distortions of sibilant and lingual–alveolar phonemes resulting from the enlarged anterior oral space and altered tongue–incisor relationship. Chewing efficiency is consistently reduced due to the absence of anterior occlusal guidance, leading to increased masticatory cycles and impaired bolus comminution. Airway dysfunction, particularly habitual mouth breathing and upper airway obstruction, contributes to abnormal tongue posture and craniofacial adaptations that maintain the open bite. These interrelated functional disturbances reinforce each other, forming a self-sustaining cycle of impaired orofacial neuromuscular activity. Early multidisciplinary intervention is therefore essential to restore normal function and promote stable occlusal development [5].

Anterior Open Bite (AOB) frequently originates from nasopharyngeal obstruction, inducing chronic oral respiration and a low-anterior tongue posture that impedes incisor eruption. This altered posture removes the tongue’s lateral expansive force against the maxillary arch while permitting unopposed buccinator muscle pressure. The resulting net inward force leads to transverse maxillary deficiency, clinically termed posterior constriction. Consequently, the narrower maxillary arch occludes lingual to the mandibular arch, establishing a posterior crossbite [6, 7]. Moreover, the absence of incisal and canine guidance during various mandibular motions may result in the wear of molar cusps. These functional issues can induce temporomandibular dysfunction [8]. Open bite has a complex etiology, encompassing hereditary and environmental factors [9]. Environmental factors include using pacifiers, tongue thrust, anterior resting posture of the tongue, mouth breathing [9], and infantile swallowing.

Anterior open bite is considered a multifactorial condition influenced by both functional and skeletal factors. Among the important etiologic contributors is upper airway obstruction, commonly associated with adenoidal and/or tonsillar hypertrophy. Enlargement of these tissues promotes habitual mouth breathing, which alters the resting posture of the tongue and affects the vertical position of the mandible. This functional adaptation is associated with downward and backward mandibular rotation, increased lower anterior facial height, and reduced vertical eruption of the maxillary incisors. Together, these changes contribute to the development and persistence of anterior open bite during growth. Recognition of airway-related influences is therefore essential when determining appropriate early intervention strategies [10]. 

Early management of AOB during the mixed dentition stage allows clinicians to positively influence facial growth patterns and oral function before skeletal discrepancies become more pronounced. Intervening at this developmental window not only facilitates more effective correction of the malocclusion but also supports improved facial esthetics and reduces psychosocial stress often associated with noticeable dental irregularities [11]. Furthermore, addressing AOB at this stage decreases the likelihood of requiring invasive treatments later on, promoting a more stable occlusal relationship and enhancing the patient’s overall quality of life from both a functional and aesthetic perspective.

Numerous procedures have been suggested for the early intervention to promote dentoalveolar development [12–15]. These protocols utilized bonded spurs due to their advantages, including inexpensive cost, compact size, aesthetic appeal, elimination of laboratory preparation, straightforward installation, and decreased chair time. Lingual-spur treatment effectively closes the AOB by maintaining tongue pressure away from the anterior teeth and reminding the patient to cease oral behaviors [16].

The optimal resting position of the tongue is characterized by the dorsum contacting the palate and the tip touching the palatal papilla. Consequently, any alteration in this resting position may result in an AOB, as the tongue’s resting position exerts low and consistent pressure sufficient to induce an open bite. The significant link between oro-muscular imbalance and the presence of the AOB suggests that tongue position at rest may be the primary causative factor for AOB formation [17]. Artese [17] categorized tongue posture into four positions: high, horizontal, low, and extremely low, due to its significance and detrimental impact on occlusion. The authors concluded that effective and stable AOB treatment relies on accurate identification and correction of the underlying etiology, particularly abnormal tongue posture at rest. Tailoring treatment strategies to the specific tongue posture enhances the likelihood of achieving long-term stability.

Anterior open bite is commonly related to the increase in the lower anterior facial height and the vertical growth pattern; therefore, some protocols have a synergistic therapy effect to modulate the growth pattern and the current habit. We are aware of just two studies that examined the impact of the combined chin cup and posterior bite block [18, 19] with the bonded spur appliance in growing patients with developing AOB, focusing on vertical molar control and mechanisms to restrict anterior and low tongue position.

We aimed to assess the therapeutic benefits of tongue spurs combined with a low transpalatal arch compared to tongue spurs alone in patients with AOB resulting from a defective tongue resting position during the mixed dentition period. The null hypothesis (H0) is that there is no disparity in the treatment effects of tongue spurs combined with a low transpalatal arch compared to tongue spurs alone in managing individuals with developing anterior open bite.

## Methods

No patient or public involvement in the design, conduct and reporting of this trial. This study was conducted in accordance with the ethical principles of the Declaration of Helsinki and its later amendments. The study received ethical approval from the Research Ethics Committee, Faculty of Dentistry, Alexandria University, Alexandria, Egypt on 17 January 2021 (IORG0008839, No. 0313 − 10/2021). The trial was retrospectively registered in the Pan African Clinical Trial Registry (PACTR202403582913859) on 14 March 2024 (https://pactr.samrc.ac.za/TrialDisplay.aspx?TrialID=27313). Written informed consent was obtained before enrollment. For all participants under the age of 16 years, written informed consent to participate was obtained from their parents or legal guardians. This study was conducted and is reported in accordance with the Consolidated Standards of Reporting Trials (CONSORT) 2010 Statement [20]. A completed CONSORT checklist is included as Additional File to support transparency and reproducibility of the reported trial. The study started in February 2021 and ended in June 2024.

In this parallel two-arm design with a balanced allocation ratio (1:1), Group I (experimental Group) received bonded Tongue Spurs in conjunction with a Low Trans-Palatal Arch (TS/LTPA), Group II (positive control group) was treated using conventional bonded Tongue Spurs alone (TS). No modifications to the methods were made after the trial began. CONSORT Statement adaptations for orthodontic trials were followed throughout the materials and techniques, results, and discussion sections to produce high-quality evidence with minimum bias [20]. 

### Sample size calculation

The sample size was calculated using G*Power version 3.1 [21] based on detecting a minimum clinically important difference of 1.5 mm in overbite correction between the two groups, using a two-tailed Mann–Whitney U test equivalent with an alpha level of 0.05 and a power of 80%. The expected standardized effect size (d ≈ 0.90) was derived from previously published randomized clinical trials evaluating tongue spur–based therapy in the mixed dentition. This calculation indicated that 32 participants (16 per group) were required, and this target sample size was achieved.

### Participants, eligibility criteria, and settings

Participants were enrolled from the Outpatient Clinic of the Department of Orthodontics, Faculty of Dentistry, Alexandria University. All participants received information about the study procedures and were shown animated videos to illustrate the steps involved. All participants exhibited a skeletal Class I relationship, confirmed clinically and cephalometrically. Children aged 7 to 11 years presenting with an anterior open bite of at least 1 mm were eligible for inclusion. Dental inclusion criteria required the presence and full eruption of the permanent first molars in both arches, fully erupted maxillary and mandibular permanent central incisors, and sufficient eruption of the lateral incisors to distinguish incomplete eruption from a true open bite. Mild anterior crowding (≤ 3 mm) was permitted, and no participant required maxillary expansion at baseline. All enrolled children demonstrated an anterior or low tongue-resting posture contributing to the open bite. The vertical relationship between the lateral and central incisors was used to differentiate incomplete eruption from a true open bite in younger patients, following the method described by Fouda et al. (2022) [22]. Children were excluded if they had a history of orthodontic treatment, craniofacial anomalies or syndromes, congenitally missing teeth, loss of permanent teeth, severe dental crowding, maxillary constriction, or posterior crossbite. Children with upper airway obstruction due to adenoidal or tonsillar hypertrophy were also excluded.

### Randomization and blinding

The random allocation sequence was generated by an independent researcher who was not involved in patient recruitment or treatment, using a permuted block randomization technique with variable block sizes to ensure balance between groups [23]. To maintain allocation concealment, the sequence codes were placed into sequentially numbered, sealed, opaque, and tamper-proof envelopes prepared by the same independent researcher. At the time of enrollment, the clinician responsible for assigning participants opened the next envelope in sequence, ensuring that neither the recruiter nor the treating operator had prior knowledge of group allocation [24]. Due to the nature of the interventions, blinding of participants and the principal operator was not feasible. However, the statistician who performed the data analysis was fully blinded to group assignment and received anonymized datasets with coded group labels to minimize assessment bias [25]. Additionally, digital cephalometric and cast analyses were performed using standardized protocols, and the measurements were recorded twice by the same examiner to enhance intra-examiner reliability.

### Participant flow

**Fig. 1:**
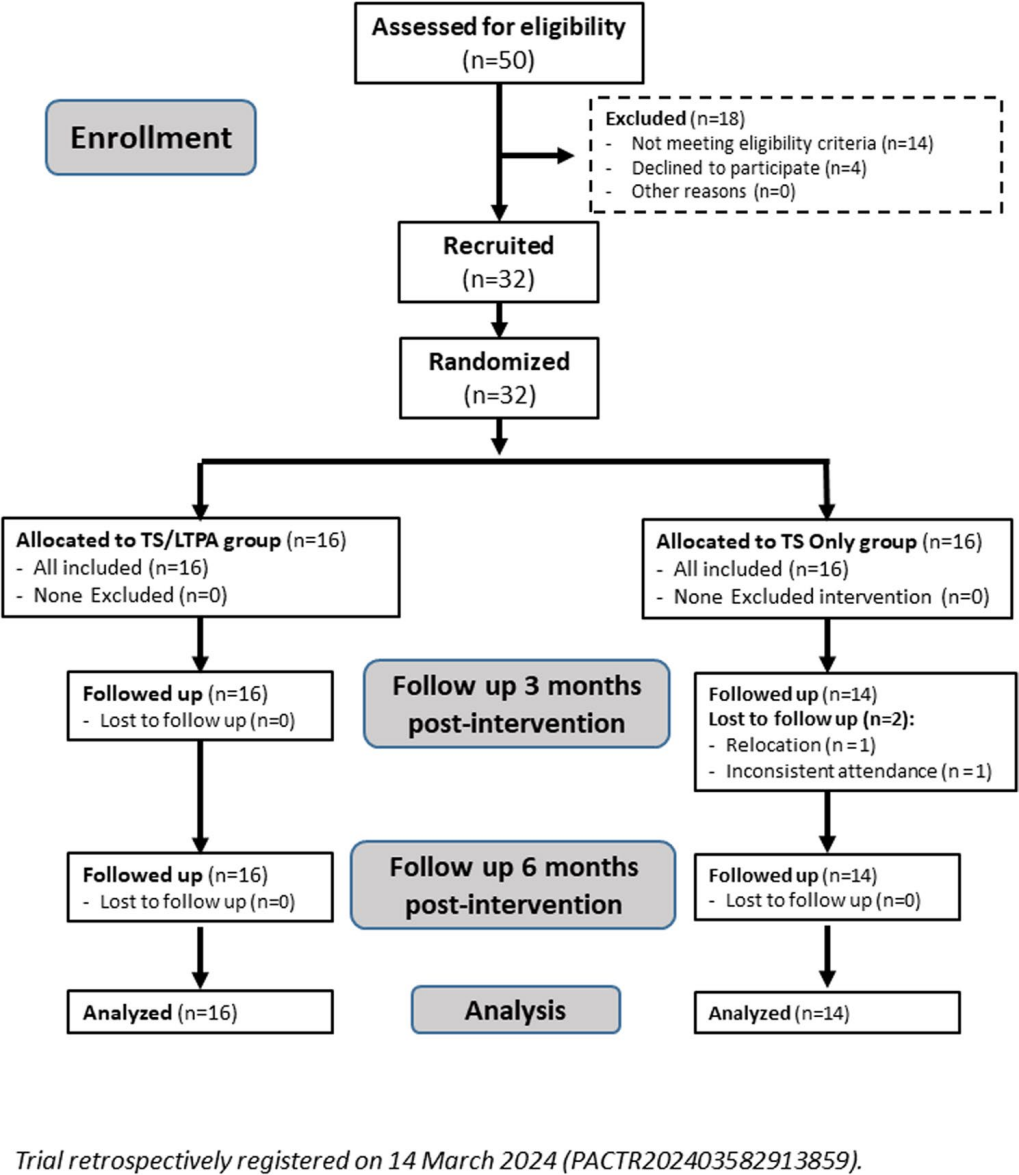
Consort flow diagram

A total of 50 children were initially assessed for eligibility according to specific inclusion and exclusion criteria. Based on these criteria, 18 children were excluded, resulting in a final sample of 32 eligible participants. These were randomly assigned into two intervention groups: the TS/LTPA group (*n* = 16) and the TS group (*n* = 16) with allocation ratio 1:1. During the follow-up period, 2 participants from the TS group discontinued the study or were lost to follow-up. These losses occurred due to relocation to another city or clinic and inconsistent attendance; no imputation was performed for missing outcome data. Accordingly, the final analyzed sample consisted of 30 patients: 16 in the TS/LTPA group and 14 in the TS group (Fig. 1).

#### Statistical methodology

The Statistical Package for Social Science (SPSS) program (ver 25)[26] was used. Shapiro–Wilk test of normality showed that most of the continuous variables were not normally distributed, so the non-parametric statistics were adopted.[27] Although age was not normally distributed, mean ± standard deviation (SD) is reported in Table 1 in accordance with journal guidelines. All inferential analyses were conducted using nonparametric tests based on medians and interquartile ranges. Data were described using minimum, maximum, median, 95% CI of the median, and 25th to 75th percentile (Inter-Quartile Range). Mann-Whitney U test[28] was used for inter-group (between-group) comparison. Wilcoxon Signed-Ranks test[29] was used for intra-group (within-group) comparison. During sample size calculation, the beta error was accepted up to 20% with a power of the study of 80%. An alpha level was set to 5% with a significance level of 95%. Statistical significance was tested at *p* value <.05. Because the trial included one prespecified primary outcome and multiple secondary outcomes, the analyses of secondary variables were considered exploratory and no formal adjustment for multiple testing was applied[20].

**Table 1:** Age and sex the studied groups

	Group		Test of significance *p*-value
	Trans palatal Arch and Lingual Tongue Spurs Group (*n* = 16)	Lingual Tongue Spurs Group (*n* = 14)	
Age (years)			
- Min. – Max. - Mean ± S.D. - Median - 95% CI of the median - 25th Percentile – 75th Percentile	7.20–11.00 8.73 ± 1.00 8.85 8.25–9.20 8.23–9.30	7.70–10.00 9.03 ± 0.68 9.25 8.60–9.60 8.60–9.60	Z_(MW)_ = 1.504 *p* =.133 NS
Sex			
- Male (*n* = 10) (33.33%) - Female (*n* = 20) (66.67%)	7 (43.75%) 9 (56.25%)	3 (21.43%) 11 (78.57%)	χ^2^_(FE)(df=1)_ = 1.674 *p* =.260 NS

*n* Number of patients, *Min-Max *Minimum – Maximum, *S.D. *Standard deviation, *CI *Confidence interval, *Z(MW) *Z test of the Mann-Whitney U test, χ2 Pearson Chi-Square, *FE *Fisher’s Exact test, *df *degree of freedom, *NS *Statistically not significant (*p*≥.05)

#### Handling of missing data and analysis population

Two participants in the TS group were lost to follow-up because of relocation to another city or clinic and inconsistent attendance that prevented completion of treatment progression. As a result, these participants did not have post-treatment cephalometric radiographs or digital models available for outcome assessment. Because the missingness occurred at the outcome-measurement level and the variables involved cannot be reliably imputed, an intention-to-treat (ITT) analysis was not feasible. Therefore, a complete-case per-protocol analysis was performed. The attrition rate was low (6.25%) and did not meaningfully affect group balance or the precision of the estimates, in accordance with CONSORT recommendations for handling missing outcome data [20].

### Interventions

Prior to appliance placement, all patients received professional scaling and polishing. Standardized oral hygiene instructions were provided using the tell-show-do method, emphasizing the use of a soft-bristled toothbrush and interdental cleaning aids to manage plaque around the spurs and arch. Patients were instructed on the use of a fluoride mouthwash and advised to report any appliance breakage immediately. Compliance was monitored and reinforced at follow-up appointments.

Bonded spurs were placed on the mid-third of the palatal surfaces of the maxillary incisors and the lingual surfaces of the mandibular incisors. These specific locations were chosen to avoid any occlusal interference throughout treatment [30]. The spurs (Matt Orthodontics, LLC, Chicago, USA) were attached using Transbond XT (3 M Unitek, Monrovia, California, USA), a light-cure orthodontic adhesive, and their tips were refined using a carborundum disk [18] before bonding. The TS/LTPA group included patients who received bonded spurs in conjunction with a low transpalatal arch. The TPA featured a loop covered with an acrylic pad positioned 8 mm from the palatal surface and was soldered to bands fitted on the maxillary permanent molars [31] (fabricated at the orthodontics Lab, Faculty of Dentistry, Alexandria University). The TS group consisted of patients who received only bonded spurs as treatment. The follow-up period spanned six months, consistent with the duration reported in similar previous studies [32, 33]. In the TS/LTPA group, the low transpalatal arch was retained after acquiring the second set of digital lateral cephalometric radiographs to facilitate long-term follow-up. Bonded spurs were maintained in both groups as active retention. Digital lateral cephalometric radiographs were taken for all participants at baseline and after six months of treatment. Alginate impressions of the upper and lower arches were obtained for each patient, along with a wax bite registration.

Impressions were immediately poured using extra-hard dental stone. The resulting stone models were scanned with the 3Shape R500 laser scanner (3Shape A/S, Copenhagen, Denmark) to generate stereolithographic (STL) files of the digital models at both the pre-treatment stage and after six months of treatment for each patient. All cephalometric analyses and superimpositions were carried out using WebCeph™ software. AOB was considered corrected when the overbite measured zero or more, with zero indicating an end-to-end vertical relationship of the incisors [34]. This was cephalometrically verified as the vertical distance, in millimeters, between the incisal edges of the maxillary and mandibular central incisors, measured perpendicular to the functional occlusal plane [16, 30]. No changes to the outcome measures were made after the trial began. All digital model measurements were conducted using 3Shape OrthoAnalyzer™ software (3Shape A/S, Copenhagen, Denmark).

#### Cephalometric analysis: (Fig. 2 A)

Craniofacial changes were evaluated using lateral cephalometric radiographs (Orthophos XG DS/ceph, Sirona), all captured with the same machine following a standardized protocol to ensure consistency across images. The cephalometric analysis included both angular and linear measurements based on 20 anatomical landmarks, as outlined by Rakosi [35]. In addition, measurements of the alveolar and basal heights of the maxilla and mandible, as described by Beckmann et al. [36], were utilized to assess changes in the anterior dentoalveolar regions [36]. To reduce measurement variability, the principal investigator conducted the radiographic analysis twice using WebCeph™ software (AssembleCircle Co. Ltd., Seoul, South Korea).

**Fig. 2:**
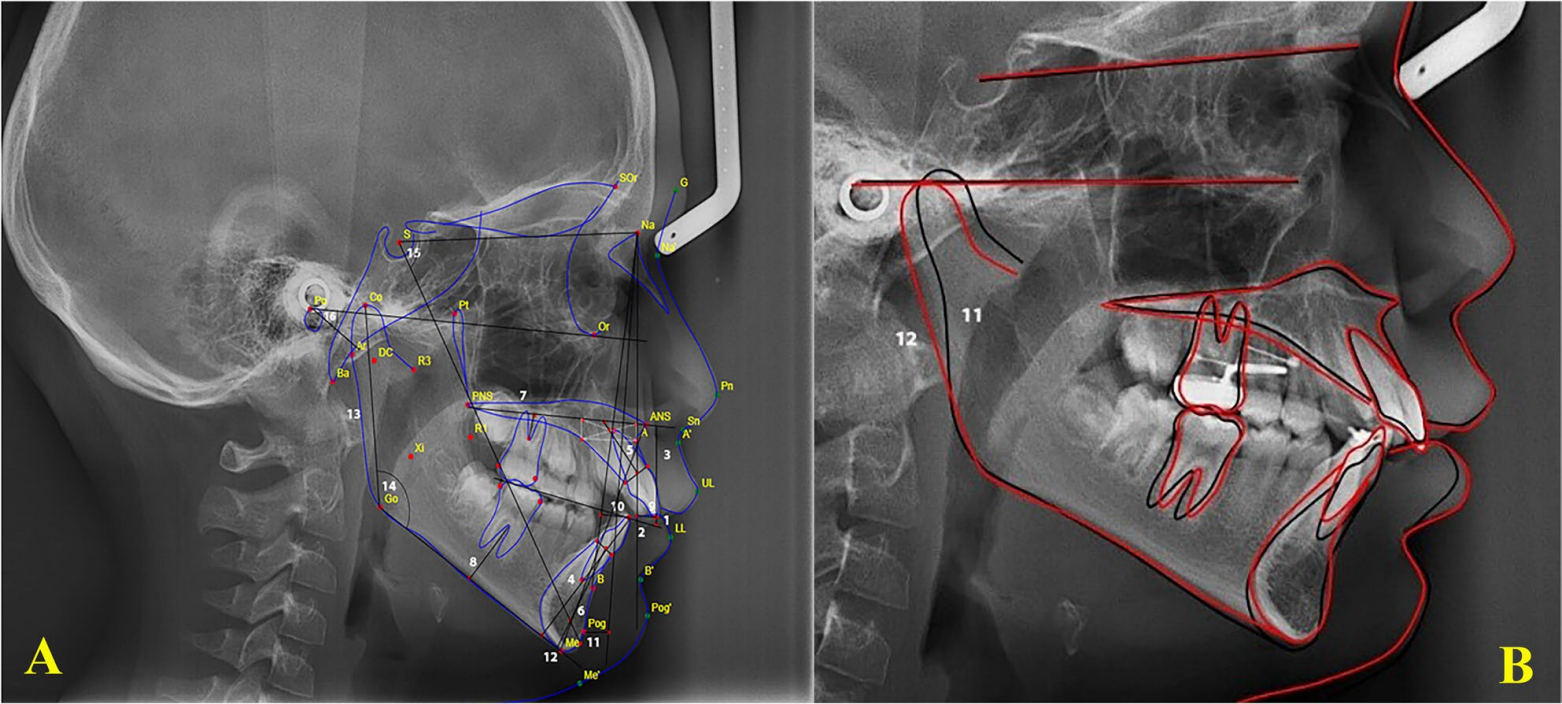
**A**: Cephalometric landmarks, linear and angular measurements(1) Overbite: Vertical distance between the incisal edges of the maxillary and mandibular central incisors, measured perpendicular to the occlusal plane.(2) Overjet: Horizontal distance between the incisal edges of the maxillary and mandibular central incisors, measured tangent to the occlusal plane.(3) U1–PP (mm): Perpendicular distance from the tip of the maxillary central incisor to the palatal plane.(4) L1–MP (mm): Perpendicular distance from the mandibular incisor tip to the mandibular plane (Go–Me).(5–6) MxH and MdH (mm): Alveolar and basal heights of the maxilla (MxH) and mandible (MdH), respectively, measured according to the method described by Beckmann et al[36].(7) U6–PP (mm): Vertical distance from the trifurcation point of the maxillary first molar to the palatal plane.(8) L6–MP (mm): Vertical distance from the bifurcation point of the mandibular first molar to the mandibular plane (Go–Me).(9) U1–NA (mm): Linear distance from the most labial point of the maxillary central incisor to the NA line.(10) L1–NB (mm): Linear distance from the most labial point of the mandibular central incisor to the NB line.(11) (FH–N)–Pg (mm): The perpendicular linear distance from Pogonion (Pg) to a line dropped perpendicularly from Nasion (N) onto the Frankfort Horizontal (FH) plane.(12) Lower Anterior Facial Height (mm): Vertical distance from the anterior nasal spine (ANS) to Menton (Me).(13) Mandibular Ramus Height (mm): Linear distance from Condylion (Co) to Gonion (Go).(14) Gonial Angle (°): Angle formed between the posterior border of the mandibular ramus and the mandibular base (Go–Ar–Me).(15) Y-Axis (°): Angle between the Sella–Gnathion (S–Gn) line and the Frankfort Horizontal plane.(16) Frankfort–Mandibular Plane Angle (FMA, °): Angle between the Frankfort Horizontal plane and the mandibular plane (Go–Me). **B**: Lateral cephalometric superimpositionPre-treatment lateral cephalometric tracing (black line).Post-treatment lateral cephalometric tracing (red line).

#### Lateral cephalometric X-ray superimposition: (Fig. 2B)

The principal investigator used WebCeph™ software, which integrates artificial intelligence, to identify and digitize all cephalometric landmarks. All linear and angular measurements were AI-assisted, and superimpositions of pre- and post-treatment cephalometric radiographs were carried out on the SN plane. To evaluate intra-observer reliability and reduce measurement variability, the principal investigator repeated the radiographic analysis and superimposition twice using the same software. To assess intra-examiner reliability, 20% of the cephalometric radiographs and digital casts were re-measured after two weeks. Intraclass correlation coefficients (ICC, two-way mixed model, absolute agreement) demonstrated excellent reliability for all variables (ICC = 0.91–0.98).

#### Digital cast analysis: ([22] (Fig. 3)

To evaluate the dentoalveolar effects of the spurs, plaster models were obtained from all 30 patients at two time points: before treatment (T1) and immediately after therapy (T2). The principal investigator conducted digital measurements on these models using the OrthoAnalyzer™ module (3Shape software), ensuring high precision. A total of 12 measurements were included in the cast analysis. Overbite was assessed as the vertical linear distance from the mesiodistal midpoint of the incisal edge of the most vertically erupted maxillary central incisor to a horizontal reference plane projected onto the corresponding mandibular central incisor.

**Fig. 3:**
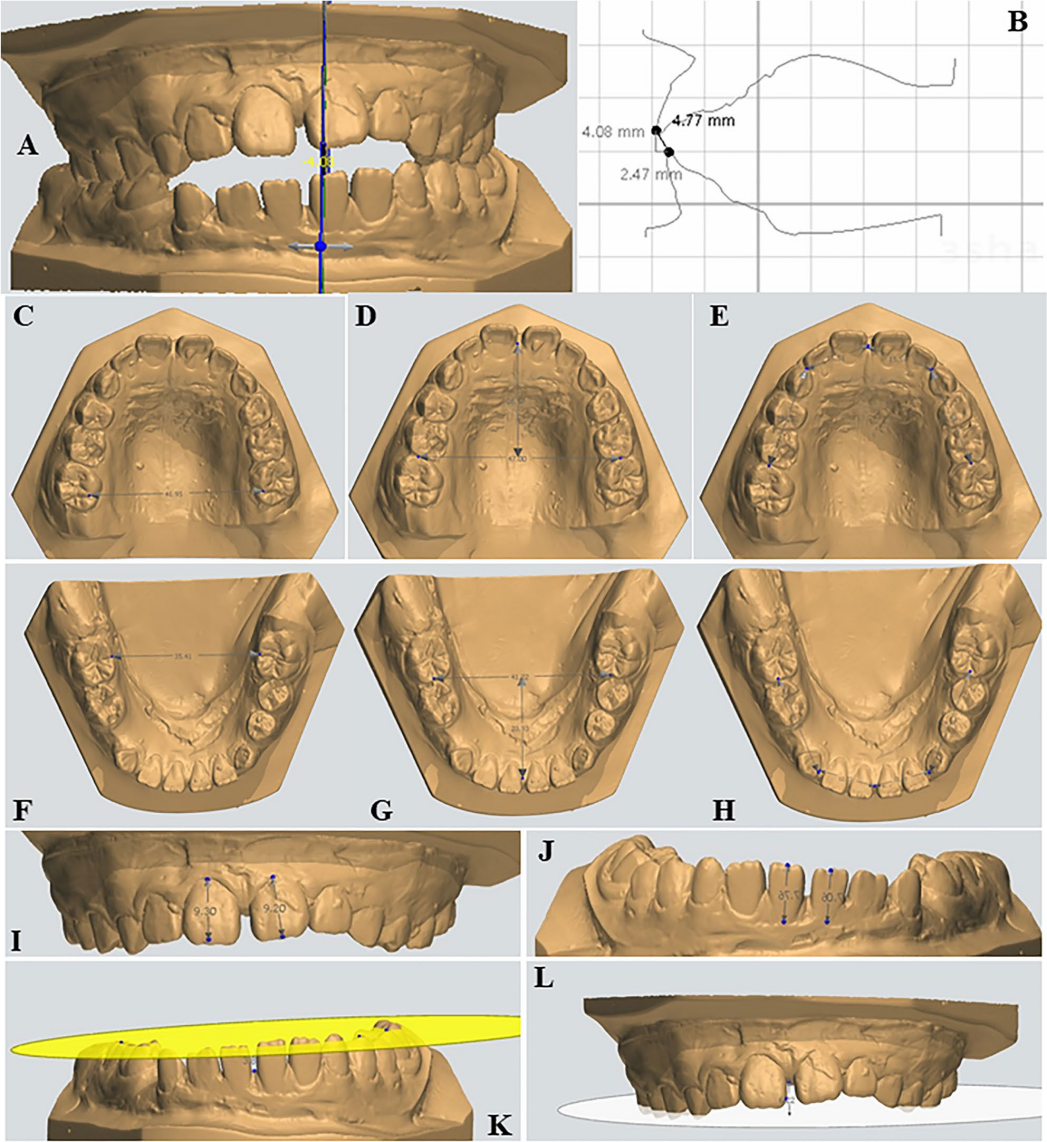
Digital cast analysis performed using 3Shape OrthoAnalyzer™ software. **A**, **B** Overbite and overjet. **C** Upper intermolar width. **D** Upper arch length. **E** Upper arch perimeter. **F** Lower intermolar width. **G** Lower arch length. **H** Lower arch perimeter. **I**, **J** Clinical crown height (upper and lower). **K**, **L** Dentoalveolar development (lower and upper arches).

The vertical dimensions of the upper and lower anterior dentoalveolar regions were determined by measuring the perpendicular distance from a point situated between the central incisors at the level of the alveolar crest to the occlusal plane, as viewed from the frontal perspective. The occlusal plane was defined using three reference points: the mesiobuccal cusp tips of the right and left permanent first molars, and the mesiobuccal cusp tip of the right primary first molar or first premolar.

Intra-arch measurements comprised arch length, arch perimeter, clinical crown height, and the anterior and posterior widths of the dental arches. After 6 months of treatment, the patient’s acceptance of the bonded spurs and the low transpalatal arch was evaluated using a structured questionnaire. The questionnaire assessed patient responses related to speech, chewing, and eating, as well as any tongue discomfort and the duration required for adaptation to the spur and low transpalatal arch therapy. The questionnaire and its findings on patient acceptance were previously published in the Egyptian Orthodontic Journal [37]. 

### Cast superimposition (Fig. 4)

Three-dimensional analysis was performed with the ortho analyzer module (3 Shape software) to superimpose the pre- and post-treatment casts for the clear visualization of the dental changes.

**Fig. 4:**
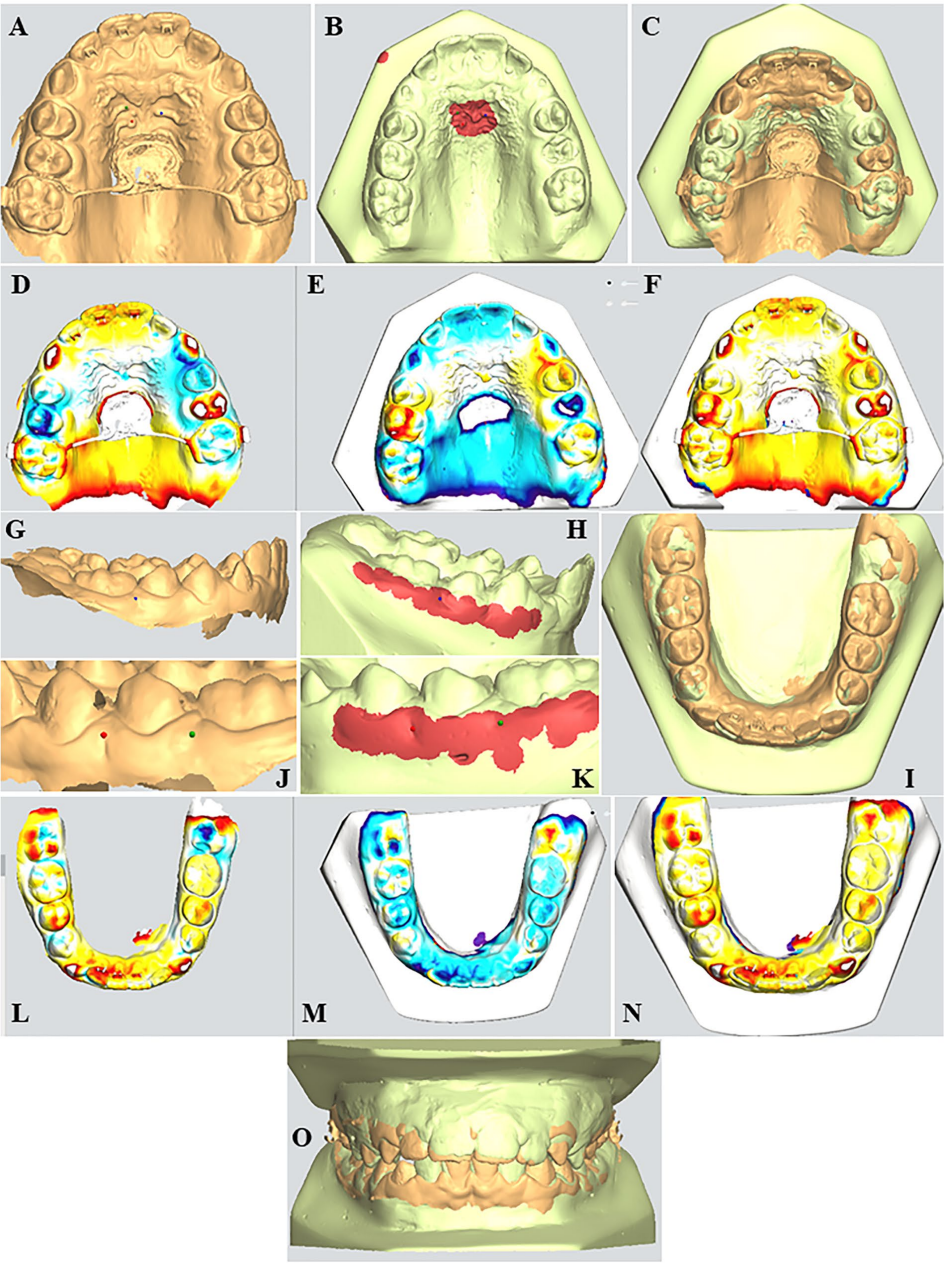
Digital cast superimposition and registration method. Superimposition was performed using a surface-based three-point registration protocol. Maxillary superimposition: (**A**,**B**) Landmarks were placed at the medial ends of the second and third palatal rugae. The defined stable reference region was bounded anteriorly by the medial two-thirds of the third rugae and laterally by lines drawn parallel to the midpalatal suture. **C** Maxillary arch superimposition (**D**-**F**) Heat map visualizations illustrating maxillary arch changes. **G**,**H**,**J**,**K** Landmarks were placed at the Muco-Gingival Junction (MGJ) between the first and second premolar and the first molar and second premolar and first molar in the contralateral side, a1 mm margin above and below the MGJ points was included to enhance stability. **I** Mandibular arch superimposition. **L**–**N** Heat map visualizations showing mandibular arch changes. **O** Occlusal overlap superimposition showing pre- and post-treatment dental alignment and occlusal changes.

### Maxillary and mandibular superimposition ([38]

Model registration was performed using the surface 3-point method, which involved selecting three corresponding landmarks on each model and marking a stable reference area for surface-based alignment. In the maxillary arch, the three points were placed on the medial ends of the second and third palatal rugae, and the reference area included the palate region bounded anteriorly by the medial two-thirds of the third rugae and laterally by two lines parallel to the mid-palatal suture. In the mandibular arch, three anatomical landmarks were initially designated along the mucogingival junction: (1) between the first and second premolars, (2) between the second premolar and first molar, and (3) between the first and second molars. A reference area was defined as a 1 mm zone extending above and below these landmarks along the mucogingival line. However, due to the developmental stage of the dentition in the present study’s sample, the mandibular second molars had not yet erupted. Consequently, the third reference point was relocated to the contralateral side, positioned between the second premolar and first molar, to ensure consistency in landmark identification and reference area selection.

No changes in the methodology after trial commencement.

### Outcomes

The primary outcome of this trial was cephalometric overbite correction. All other cephalometric, skeletal, soft-tissue, and digital cast variables were considered exploratory secondary outcomes.

### Ethics approval and consent to participate

The study was approved by the institutional review board at the Faculty of Dentistry, Alexandria University, Alexandria, Egypt, under international number: IORG0008839 and ethics committee no:0313-1/2021 on 17 January 2021. Written informed consent was obtained before enrollment. For all participants under the age of 16 years, written informed consent to participate was obtained from their parents or legal guardians. This study was conducted in accordance with the ethical principles of the Declaration of Helsinki and its later amendments.

## Results

### Demographic data (Table 1)

The baseline demographic and clinical parameters, including age, sex distribution, AOB severity, craniofacial growth pattern, and initial cephalometric variables, were comparable. A notable observation was the predominance of female participants in the intervening groups, with a female: male ratio of approximately 2:1. The mean age was 8.73 ± 1.00 years in the TS/LTPA group and 9.03 ± 0.68 years in the TS group, with no significant difference between groups (*p* =.133).

### Analysis of population and missing data

During the study, 2/16 (12.5%) participants from the TS group were lost to follow-up. As the primary analysis was performed per protocol, data from 16 patients in the TS/LTPA Group and 14 patients in the TS group were analyzed. The attrition rate did not compromise the statistical power or confidence intervals.

### Treatment outcomes (Figs. 5 and 6)

**Fig. 5:**
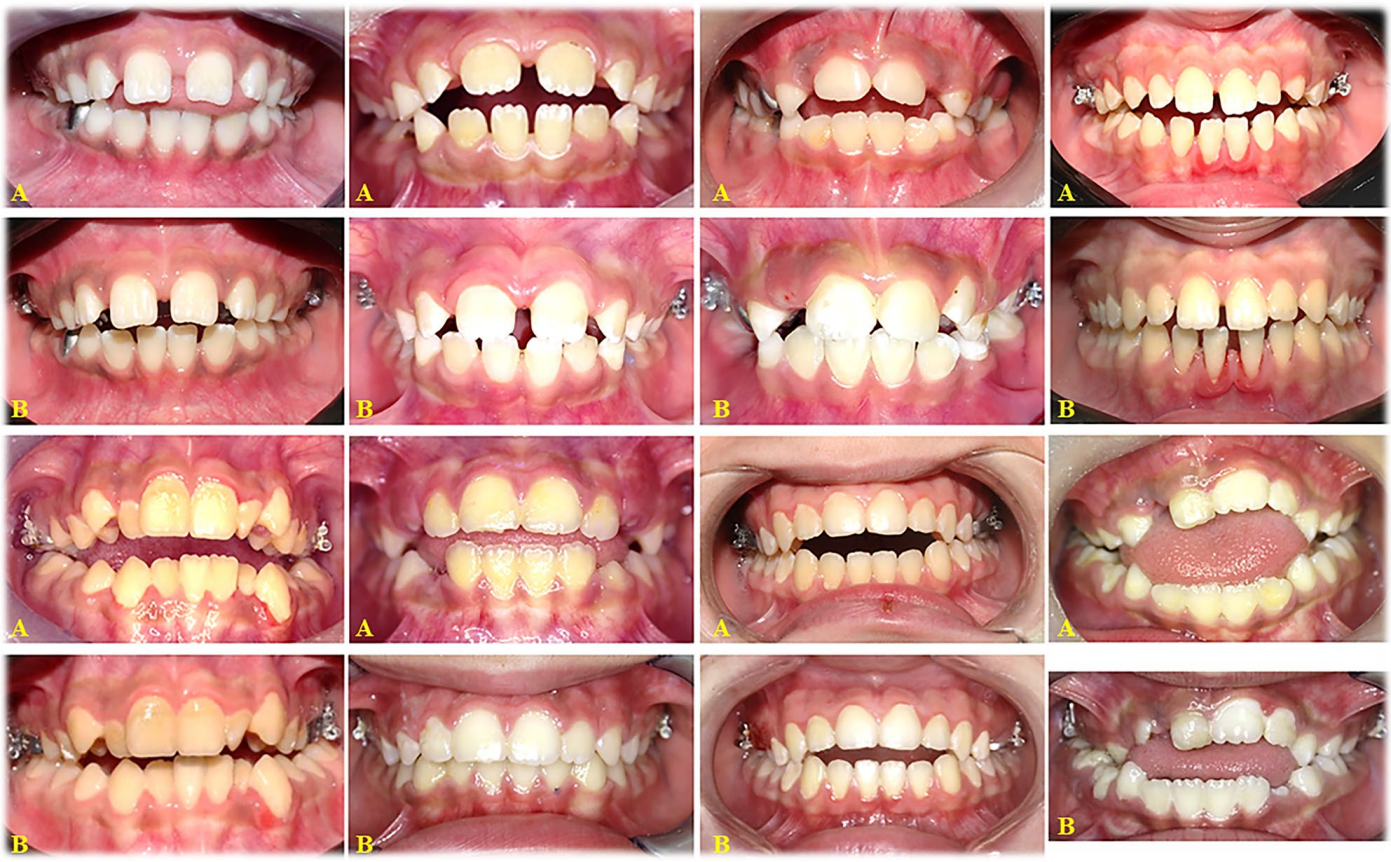
Intraoral photographs of developing open bite closure in the (TS/LTPA) Group. **A** Pre-treatment. **B** Post-treatment

**Fig. 6:**
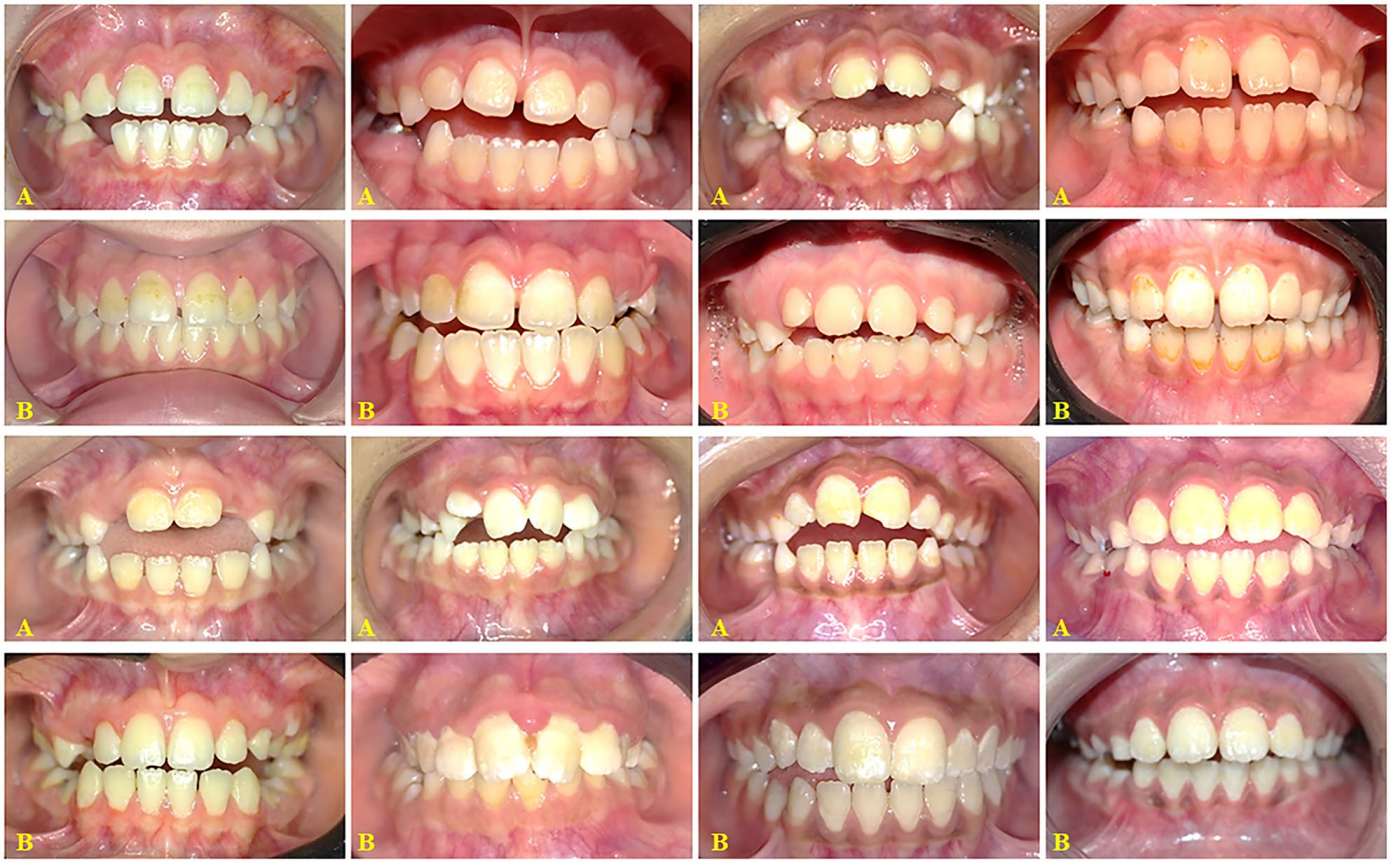
Intraoral photographs of developing open bite closure in the (TS) group. **A** Pre-treatment. **B** Post-treatment

#### Cephalometric analysis

##### Dental changes: (Table 2)

Both groups demonstrated statistically significant improvement in AOB after six months of intervention, with no significant intergroup differences. Positive overbite was observed in 6 of 16 patients (37.5%) in the TS/LTPA group and 8 of 14 patients (57.1%) in the TS group. The median overbite change correction was 3.35 mm and 3.90 mm for the TS/LTPA and TS groups, respectively, without a statistically significant difference between them.

**Table 2:** Pretreatment and post-treatment cephalometric variables (dental readings) in the studied groups

	Trans palatal Arch and Lingual Tongue Spurs Group			Lingual Tongue Spurs Group		
	T1 (*n* = 16)	T2 (*n* = 16)	Change (*n* = 16)	T1 (*n* = 14)	T2 (*n* = 14)	Change (*n* = 14)
Overbite (cephalometric) (mm)						
Median (IQR) 95% CI of the median	− 5.20^ab^ (− 8.40 - −3.10) − 7.50 - −3.20	− 1.75^ab^ (− 5.60 − 0.50) − 5.00–0.00	3.35^ab^ (1.05–5.68) 1.10–5.60	− 3.75^ab^ (− 5.70 - −3.20) − 5.70 - − 3.20	0.00^ab^ (− 1.00–0.50) − 0.40–1.00	3.90^ab^ (2.80–5.70) 2.90–6.70
* p* - value	*p* <.001*			*p* <.001*		
Overjet (cephalometric) (mm)						
Median (IQR) 95% CI of the median	4.88^ab^ (4.32–6.60) 4.59–6.20	5.87^ab^ (3.88–6.50) 4.75–6.00	0.07^ab^ (− 1.00–0.88) − 1.00–0.81	4.40^ab^ (3.00–6.00) 3.00–6.00	4.00^ab^ (2.00–6.00) 2.00–6.00	− 1.00^ab^ (− 1.00 - − 1.00) − 1.00–4.56
* p* - value	*p* =.777 NS			*p* =.046*		
Upper first molar vertical position (mm)						
Median (IQR) 95% CI of the median	7.95^ab^ (7.05–9.10) 7.10–9.10	8.35^ab^ (7.45–9.45) 7.50–9.40	0.40^a^ (0.30–0.65) 0.40–0.70	7.95^ab^ (1.00–8.50) 7.00–8.50	8.90^ab^ (8.00–9.50) 8.00–9.60	0.90^b^ (0.70–1.00) 0.80–1.00
* p* - value	*p* =.001*			*p* =.001*		
Lower first molar vertical position (mm)						
Median (IQR) 95% CI of the median	15.20^ab^ (14.20–16.80) 15.00–16.80	15.80^ab^ (14.80–17.60) 15.20–17.30	0.50^a^ (0.40–0.65) 0.50–0.90	14.20^ab^ (12.10–15.90) 12.10–15.90	14.30^ab^ (12.30–15.90) 12.30–15.90	0.15^b^ (0.00–0.20) 0.00–0.20
* p - value*	*p* =.001*			*p* =.004*		
Upper first molar angulation (◦)						
Median (IQR) 95% CI of the median	99.50^ab^ (96.00–106.00) 96.00–105.00	98.00^ab^ (94.00–102.50) 94.00–102.00	− 2.00^ab^ (− 8.50–1.50) − 7.00–2.00	100.50^ab^ (96.00–103.00) 96.00–103.00	101.00^ab^ (97.00–109.00) 97.00–109.00	− 0.50^ab^ (− 1.00–2.00) − 1.00–2.00
* p* - value	*p* =.157 NS			*p* =.843NS		
Upper central/Frankfort (◦)						
Median (IQR) 95% CI of the median	116.00^ab^ (113.00–118.00) 114.00–118.00	114.00^ab^ (110.00–118.00) 110.00–118.00	− 1.00^ab^ (− 6.00–0.00) − 2.00–0.00	118.50^ab^ (116.00–122.00) 116.00–122.00	116.50^ab^ (114.00–118.00) 114.00–118.00	− 2.00^ab^ (− 4.00–0.00) − 4.00–0.00
* p* - value	*p* =.020*			*p* =.026*		
Lower central/mandibular plane (◦)						
Median (IQR) 95% CI of the median	104.5^ab^ (96.00–109.00) 97.00–109.00	95.50^ab^ (96.00–101.00) 91.00–104.00	− 4.00^ab^ (− 6.00 - −3.00) − 5.00 - −3.00	98.50^ab^ (96.00–101.00) 96.00–101.00	97.50^ab^ (92.00–100.00) 92.00–100.00	− 1.00^ab^ (− 4.00–0.00) − 2.00–0.00
* p* - value	*p* =.001*			*p* =.041*		
Interincisal angle (◦)						
Median (IQR) 95% CI of the median	105.50^a^ (103.50–113.00) 104.00–112.00	112.00^ab^ (11.00–114.00) 109.00–117.00	5.50^ab^ (3.00–7.00) 3.00–6.00	112.00^b^ (110.00–114.00) 110.00–114.00	116.00^ab^ (113.00–125.00) 113.00–125.00	5.00^ab^ (2.00–7.00) 2.00–7.00
* p* - value	*p <*.001*			*p* =.002*		
U1 - PP (mm)						
Median (IQR) 95% CI of the median	22.50^ab^ (20.30–24.35) 20.60–23.90	23.65^ab^ (21.10–24.80) 21.30–24.60	0.80^ab^ (0.65–1.45) 0.70–1.50	22.70^ab^ (20.50–24.30) 20.50–24.30	23.85^ab^ (21.00–24.90) 21.00–24.90	0.95^ab^ (0.60–1.00) 0.60–1.00
* p* - value	*p* =.001*			*p* =.001*		
L1 - MP (mm)						
Median (IQR) 95% CI of the median	32.10^ab^ (30.10–34.55) 32.00–36.10	33.00^ab^ (31.20–36.01) 32.30–37.00	0.95^ab^ (0.85–1.45) 0.90–1.40	32.35^ab^ (31.40–35.70) 31.40–35.70	33.5^ab^ (32.50–36.00) 32.50–36.00	0.95^ab^ (0.50–1.10) 0.50–1.10
* p* - value	*p* <.001*			*p* =.001*		
Upper lip to E Line (mm)						
Median (IQR) 95% CI of the median	1.33^ab^ (− 3.12–3.41)	0.93^ab^ (− 1.04–2.60)	− 0.95^ab^ (− 2.18–2.44)	2.00^ab^ (− 2.00–4.00)	− 1.50^ab^ (− 4.00–2.00)	− 2.00^ab^ (− 2.00 - −1.00)
* p* - value	*p* =.717 NS			*p* =.003*		
Lower Lip to E Line (mm)						
Median (IQR) 95% CI of the median	4.77^ab^ (− 0.75–7.18) − 0.50–7.00	3.01^ab^ (− 0.80–6.50) − 0.60–6.00	− 2.01^ab^ (− 3.40 - −0.75) − 3.00 – − 1.00	5.00^ab^ (1.00–7.00) 1.00–7.00	2.00^ab^ (− 1.00–5.00) − 1.00–5.00	− 2.00^ab^ (− 4.00 - −1.00) − 2.00–1.30
* p* - value	*p* =.046*			*p* =.015*		
Nasolabial Angle (◦)						
Median (IQR) 95% CI of the median	101.50^ab^ (91.00–106.50) 91.00–103.00	105.50^ab^ (98.50–108.00) 99.00–108.00	5.00^ab^ (3.00–9.50) 3.00–9.00	102.50^ab^ (92.00–108.00) 92.00–108.00	104.00^ab^ (94.00–113.00) 94.00–113.00	4.50^ab^ (3.00–6.00) 3.00–6.00
* p* - value	*p* =.011*			*p* =.010*		
Upper Central NA Line (mm)						
Median (IQR) 95% CI of the median	6.10^a^ (4.80–7.60) 5.00–7.50	5.70^ab^ (4.25–7.10) 4.40–7.00	− 0.50^a^ (− 0.90 − 0.30) − 0.60 − 0.30	4.55^b^ (3.80–6.40) 3.80–6.40	4.40^ab^ (3.60–6.00) 3.60–6.00	− 0.25^b^ (− 0.40–0.00) − 0.40–0.00
* p* - value	*p* <.001*			*p* =.005*		
Lower Central to NB Line (mm)						
Median (IQR) 95% CI of the median	7.90^ab^ (6.25–8.30) 6.80–8.40	7.00^ab^ (5.70–8.00) 6.10–8.00	− 0.50^a^ (− 0.85 - −0.40) − 0.80 - −0.40	6.48^ab^ (5.40–7.50) 5.40–7.50	6.40^ab^ (5.00–7.10) 5.00–7.10	− 0.30^b^ (− 0.40–0.00) − 0.40–0.00
* p* - value	*p* <.001*			*p* =.018*		

*n* Number of patients, *IQR *Interquartile Range (25th percentile – 75th percentile), *p *Probability of error (Wilcoxon Signed Ranks Test)

*Superscript letters (a,b) *Comparison of the two studied groups using the Mann - Whitney U test; *similar superscript at T1, T2, or change denote non statistically significant difference *

*MW* Mann - Whitney U, *WSR *Wilcoxon Signed Ranks Test

*Statistically significant (*p*<.05), *NS *Statistically significant (*p*≥.05)

Effect size for the primary outcome (Overbite change): Hedges g = –0.18 (95% CI: –0.90 to 0.53)

For the primary outcome (cephalometric overbite change), the between-group standardized effect size (Hedges g) was − 0.18 (95% CI: − 0.90–0.53), indicating a very small and clinically negligible difference between TS/LTPA and TS in overbite improvement over 6 months.

Regarding molar vertical control, the maxillary first molar vertical position (U6–PP) showed a vertical position change of + 0.40 mm (IQR: 0.30 to 0.65 mm) in the TS/LTPA group and + 0.90 mm (IQR: 0.70 to 1.00 mm) in the TS group. The differences observed between the groups were statistically significant. Similarly, the vertical position of the mandibular first molar (L6–MP) demonstrated a significantly greater increase in the TS/LTPA group, with a median change of 0.50 mm (IQR: 0.40 to 0.65 mm), compared with a median increase of 0.15 mm (IQR: 0.00 to 0.20 mm) in the TS group, yielding a statistically significant intergroup difference.

Maxillary molar angulation showed a non-significant decrease in both groups. Both maxillary incisor angulation (U1–FH (°)) and mandibular incisor angulation (L1–MP (°)) demonstrated statistically significant post-treatment reductions in both groups. For U1–FH, the TS/LTPA group showed a median decrease of − 1.00° (IQR: − 6.00 to 0.00°), while the TS group exhibited a median reduction of − 2.00° (IQR: − 4.00 to 0.00°). Regarding L1–MP, the TS/LTPA group recorded a markedly greater median decrease of − 4.00° (IQR: − 6.00 to − 3.00°) compared with − 1.00° (IQR: − 4.00 to 0.00°) in the TS group. Despite these within-group improvements, intergroup comparisons revealed no statistically significant differences.

Extrusion of both maxillary and mandibular incisors, measured by U1–PP and L1–MP, respectively, showed statistically significant increases post-treatment in both groups. For U1–PP, the TS/LTPA group exhibited a median increase of 0.80 mm (IQR: 0.65 to 1.45 mm), while the TS group showed a similar increase of 0.95 mm (IQR: 0.60 to 1.00 mm). As for L1–MP, both groups demonstrated comparable median increases of 0.95 mm; the TS/LTPA group had an IQR of 0.85 to 1.45 mm, and the TS group had an IQR of 0.50 to 1.10 mm. No statistically significant intergroup differences were observed.

Evaluation of upper incisor position relative to the NA line (U1–NA (mm)) revealed a median reduction of − 0.50 mm (IQR: − 0.90 to − 0.30 mm) in the TS/LTPA group and − 0.25 mm (IQR: − 0.40 to 0.00 mm) in the TS group, with significant intergroup difference. Similarly, the mandibular central incisors (L1–NB (mm)) showed median reductions of − 0.50 mm (IQR: − 0.85 to − 0.40 mm) and − 0.30 mm (IQR: − 0.40 to 0.00 mm) in the TS/LTPA and TS groups, respectively, with significant intergroup differences.

##### Skeletal changes (Table 3)

The (FH–N)–Pg distance significantly decreased post-intervention by 0.50° (IQR: 0.15 to 0.90°) and 0.65° (IQR: 0.00 to 2.00°) in both TS/LTPA and TS groups, respectively. However, no statistically meaningful difference was found between the two groups regarding this change. Intragroup analysis revealed a statistically significant increase in the Y-axis angle within the Tongue Spurs group, with a median change of 1.00° (IQR: 1.00 to 2.00°), whereas no significant change was detected in the TS/LTPA group. However, the intergroup comparison showed no statistically significant difference in Y-axis angle changes between the two groups.

**Table 3:** Pretreatment and post-treatment cephalometric variables (skeletal readings) in the studied groups

	Trans palatal Arch and Lingual Tongue Spurs Group			Lingual Tongue Spurs Group		
	T1 (*n* = 16)	T2 (*n* = 16)	Change (*n* = 16)	T1 (*n* = 14)	T2 (*n* = 14)	Change (*n* = 14)
SNA Angle (◦)						
- Median (IQR) - 95% CI of the median	83.00^ab^ (79.50–84.00) 80.00–84.00	83.50^ab^ (81.00–85.50.00.50) 81.00–85.00	0.50^ab^ (−0.50−2.00) 0.00–2.00	83.50^ab^ (82.00–85.00) 82.00–85.00	83.50^ab^ (80.00–87.00) 80.00–87.00	0.50^ab^ (0.00–2.00) 0.00–2.00
* p*-value	*p* =.178 NS			*p* =.383 NS		
SNB Angle (◦)						
- Median (IQR) - 95% CI of the median	75.50^ab^ (73.00–79.50.00.50) 74.00–79.00	77.50^ab^ (74.00–81.00) 74.00–80.00	1.00^ab^ (0.00–3.00) 1.00–3.00	78.50^ab^ (75.00–80.00) 75.00–80.00	77.50^ab^ (76.00–82.00) 76.00–82.00	1.50^ab^ (0.00–2.00) 0.00–2.00
* p-value*	*p* =.004*			*p* =.125 NS		
ANB Angle (◦)						
- Median (IQR) - 95% CI of the median	6.00^ab^ (4.50–8.00.50.00) 5.00–8.00	5.50^ab^ (3.50–7.50) 4.00–7.00	−1.00^ab^ (−2.00−0.00) −1.00−0.00	5.00^ab^ (4.00–6.00) 5.00–7.00	5.00^ab^ (4.00–7.00) 4.00–7.00	0.00^ab^ (−1.00−1.00) 0.00–1.00
* p*-value	*p* =.164 NS			*p* =.713 NS		
Mandibular plane angle (◦)						
- Median (IQR) - 95% CI of the median	29.00^ab^ (26.50–34.50) 27.00–33.00	28.00^ab^ (26.50–32.50) 27.00–33.00	−1.00^ab^ (−2.00−0.00) −1.00–0.00	29.50^ab^ (27.00–33.00) 27.00–33.00	28.50^ab^ (25.00–32.00) 25.00–32.00	−0.50^ab^ (−3.00−0.00) −3.00–0.00
* p*-value	*p* =.003 S			*p* =.017*		
Gonial angle						
- Median (IQR) - 95% CI of the median	122.00^ab^ (119.00–125.50.00.50) 120.00–129.00.00.00	125.00^ab^ (122.00–128.00.00.00) 122.00–128.00.00.00	1.00^a^ (−1.00−5.00) 0.00–5.00	125.00^ab^ (123.00–132.00.00.00) 123.00–134.00.00.00	122.29^ab^ (122.00–128.00.00.00) 122.00–128.00.00.00	−1.50^b^ (−4.00−1.00) −4.00−1.00
* p*-value	*p* =.083 NS			*p* =.222 NS		
LAFH (mm)						
- Median (IQR) - 95% CI of the median	55.20^ab (^51.95–56.35) 54.60–57.30	55.30^ab^ (52.65–57.00.65.00) 52.80–56.20	0.65^ab^ (0.45–0.80) 0.50–0.80	56.85^ab^ (54.50–60.00) 54.50–60.00	57.50^ab^ (55.00–61.00) 55.00–61.00	0.75^ab^ (0.50–1.00.50.00) 0.50–1.00.50.00
* p*-value	*p* =.006*			*p* =.001*		
Y-axis angle (◦)						
- Median (IQR) - 95% CI of the median	69.00^ab^ (66.50–71.50) 67.00–72.00	70.50^ab^ (66.00–72.50.00.50) 66.00–72.00	0.00^ab^ (−1.00−2.00) 0.00–2.00	70.50^ab^ (68.00–74.00) 68.00–74.00	72.00^ab^ (69.00–74.00) 69.00–75.00	1.00^ab^ (1.00–2.00) 1.00–2.00
* p*-value	*p* =.118 NS			*p* =.039*		
FH–N–Pg (◦)						
- Median (IQR) - 95% CI of the median	−4.50^ab^ (−6.65−0.75) −6.30 - −2.50	−3.90^ab^ (−5.95−0.75) −5.90- −2.00	0.50^ab^ (0.15–0.90) 0.30–1.00.30.00	−5.00^ab^ (−6.00−2.00) −5.20- −1.00	−2.25^ab^ (−5.00−1.00) −5.00- −1.00	0.65^ab^ (0.00–2.00) 0.00–2.00
* p-value*	*p* =.003*			*p* =.005*		
Effective Ramus growth (mm)						
- Median - IQR	44.25^ab^ (42.05–46.10) 42.30–45.40	45.10^ab^ (43.45–47.10) 43.90–46.80	0.75^ab^ (0.60–1.40) 0.60–1.40	43.80^ab^ (40.00–45.30.00.30) 40.00–45.30.00.30	45.15^ab^ (41.10–47.00) 41.10–47.00	1.05^ab^ (0.70–1.90) 0.70–1.90
* p-value*	*p* <.001*			*p* =.001*		
Maxillary Alveolar Height (Cephalometric) (mm)						
- Median (IQR) - 95% CI of the median	28.05^ab^ (24.10–30.35.10.35) 24.60–30.00	28.75^ab^ (24.45–31.40) 24.90–30.80	0.85^ab^ (0.50–0.95) 0.60–0.90	26.95^ab^ (23.60–28.60) 23.60–28.60	27.90^ab^ (25.00–29.20.00.20) 25.00–29.20.00.20	0.75^ab^ (0.50–1.40) 0.50–1.40
* p-value*	*p* <.001*			*p* =.001*		
Mandibular Alveolar Height (Cephalometric) (mm)						
- Median (IQR) - 95% CI of the median	44.65^ab^ (41.75–49.10) 42.10–48.60	45.25^ab^ (42.50–49.50) 43.00–49.00	0.55^ab^ (0.40–0.85) 0.40–0.80	45.15^ab^ (42.90–46.80) 42.90–46.80	45.95^ab^ (43.90–48.00) 43.90–48.00	0.85^ab^ (0.40–1.20) 0.40–1.20
* p*-value	*p* =.001*			*p* =.001*		

*n* Number of patients, *IQR* Interquartile Range (25th percentile – 75th percentile), *p*: Probability of error (Wilcoxon Signed Ranks Test),

*Superscript letters (a,b*): Comparison of the two studied groups using the Mann-Whitney U test; *similar superscript at T1, T2, or change denote non statistically significant difference *

*MW *Mann-Whitney U, *WSR *Wilcoxon Signed Ranks Test

*Statistically significant (*p*<.05), *NS* Statistically significant (*p*≥.05)

A decrease in the mandibular plane angle was observed in both treatment groups. The TS/LTPA group exhibited a median reduction of − 1.00° (IQR: − 2.00 to 0.00°), while the TS group showed a median decrease of − 0.50° (IQR: − 3.00 to 0.00°). However, the intergroup difference was not statistically significant.

Additionally, lower anterior facial height increased significantly in both groups, with median changes of + 0.65 mm (IQR: 0.45–0.80 mm) in the TS/LTPA group and + 0.75 mm (IQR: 0.50 to 1.00 mm) in the TS group, with no intergroup differences. 

Effective ramus height increased by a median of + 0.75 mm (IQR: 0.60 to 1.40 mm) in the TS/LTPA group and + 1.05 mm (IQR: 0.70 to 1.90 mm) in the TS group. Although both groups demonstrated statistically significant intragroup increases, the intergroup comparison remained non-significant (*p* >.05).

Maxillary alveolar height showed a significant increase in both groups, with median changes of 0.85 mm (IQR: 0.50 to 0.95 mm) in the TS/LTPA group and 0.75 mm (IQR: 0.50 to 1.40 mm) in the TS group, with no statistically reliable difference. Similarly, mandibular alveolar height increased significantly in both groups, 0.55 mm (IQR: 0.40 to 0.85 mm) and 0.85 mm (IQR: 0.40 to 1.20 mm), respectively, without significant intergroup difference.

##### Soft tissue changes (Table 2)

Significant changes were observed in soft tissue parameters in both groups. The nasolabial angle increased significantly, and both upper and lower lips retruded relative to the E-line. However, intergroup differences were not statistically significant.

#### Digital cast analysis (Table 4)

Digital model analysis revealed that both groups experienced a significant reduction in AOB, with comparable overbite changes of 2.93 mm (IQR: 1.16 to 5.00 mm) in TS/LTPA and 3.67 mm (IQR: 2.00 to 5.00 mm) in TS, and no significant difference between groups. The median overjet changes in the TS/LTPA group reflected a mild increase of + 0.07 mm (IQR: − 0.52 to 0.62 mm), whereas the TS group presented a more pronounced median decrease of − 0.61 mm (IQR: − 1.00 to − 0.13 mm). Pretreatment and post-treatment digital cast variables in the studied Groups

**Table 4:** Pretreatment and post-treatment digital cast variables in the studied groups

	Trans palatal Arch and Lingual Tongue Spurs Group			Lingual Tongue Spurs Group			
	T1 (*n* = 16)	T2 (*n* = 16)	Change (*n* = 16)	T1 (*n* = 14)	T2 (*n* = 14)		Change (*n* = 14)
Upper Arch Length (mm)							
- Median (IQR) - 95% CI of the median	26.48^ab^ (24.3–29.20) 24.57–28.71	25.94^ab^ (22.76–28.43) 23.00–27.88	−1.22^ab^ (−1.55 – −0.47) −1.53 – −0.70	27.41^ab^ (26.15–28.71) 26.15–28.71	26.03^ab^ (25.11–27.13) 25.11–27.13		−1.04^ab^ (−1.64 – −0.75) −1.64 – −0.75
* p-value*	*p* =.001*			*p* =.0013*			
Lower Arch Length (mm)							
- Median (IQR) - 95% CI of the median	25.25^ab^ (21.95–26.08) 24.15–26.16	23.10^ab^ (20.64–24.19) 21.53–24.16	−1.73^ab^ (−2.58 – −1.01) −2.30 – −1.04	22.85^ab^ (21.41–25.82) 21.41–25.82	21.18^ab^ (20.21–24.68) 20.21–24.68		−1.60^ab^ (−2.51 – −1.22) −2.51 – −1.22
- *p*-value	*p* <.003*			*p* =.084 NS			
Upper Arch Perimeter (mm)							
- Median (IQR) - 95% CI of the median	73.78^ab^ (70.46–78.13) 72.05–78.01	73.05^ab^ (68.08–75.84) 69.45–75.32	−1.85^ab^ (−2.54 – −0.49) −2.52 – −0.52	70.81^ab^ (67.43–76.43) 67.43–76.43	71.01^ab^ (66.00–75.55) 66.00–75.55		−1.63 (−2.47 – −0.60) −2.47 – −0.60
* p*-value	*p* =.001*			*p* =.016*			
Lower Arch Perimeter (mm)							
- Median (IQR) - 95% CI of the median	67.52^ab^ (62.11–70.16 62.69–69.90	65.11^ab^ (59.83–67.91) 60.00–67.91	−3.19^ab^ (−6.45 – −0.78) −6.40 – −2.00	68.40^ab^ (66.32–71.33) 66.32–71.33	65.04^ab^ (59.89–66.03) 59.89–66.03		−6.21^ab^ (−8.15 – −2.61) −8.15 – −2.61
* p*-value	*p* =.148 NS			*p* <.001*			
Upper Inter-Arch Distance (mm)							
- Median (IQR) - 95% CI of the median	38.70^ab^ (35.24–42.10) 35.61–41.19	38.70^ab^ (35.24–42.10) 35.61–41.19	0.00^a^ (0.00–0.00)	36.72^ab^ (33.94–38.23) 33.94–38.23	38.28^ab^ (35.30–40.00) 35.30–40.00		1.34^b^ (0.89–1.60) 0.89–1.68
* p*-value	*p* =.067 NS			*p* =.394 NS			
Upper right clinical crown (mm)							
- Median (IQR) - 95% CI of the median	8.84^ab^ (7.31–9.46) 7.48–9.38	8.86^ab^ (7.77–9.49) 8.03–9.43	0.05^ab^ (−0.08–0.30) −0.04–0.30	8.51^ab^ (7.74–9.50) 7.74–9.50	8.87^ab^ (7.66–9.93) 8.10–9.95		0.18^ab^ (−0.02–0.67) −0.02–0.67
* p*-value	*p =*.460 NS			*p* =.044*			
Lower left clinical crown (mm)							
- Median (IQR) - 95% CI of the median	6.68^ab^ (6.40–7.21) 6.40–7.20	7.28^ab^ (6.92–7.75) 7.00–7.63.00.63	0.62^ab^ (−0.03−0.83) 0.14–0.82	6.89^ab^ (6.66–7.55) 6.66–7.55	7.30^ab^ (6.55–7.77) 6.55–7.77		0.28^ab^ (−0.09–0.65) −0.09–0.65
* p*-value	*p =*.026*			*p* =.084 NS			
Lower Inter-Arch Distance (mm)							
- Median (IQR) - 95% CI of the median	33.94^ab^ (32.60–37.66) 33.00–37.21	36.58^ab^ (34.14–39.38) 34.28–38.84	1.07^ab^ (0.36–2.51) 0.53–2.01	34.74^ab^ (32.81–35.05) 32.81–35.05	36.30^ab^ (34.57–37.33) 34.57–37.33		1.30^ab^ (0.08–2.89) 0.08–2.89
* p*-value	*p* =.001*			*p* =.008*			
Maxillary Alveolar Height (Cast) (mm)							
- Median (IQR) - 95% CI of the median	6.64^ab^ (5.47–7.69) 5.64–7.23	5.25^ab^ (4.90–6.50) 5.00–6.50.00.50	−0.92^ab^ (−1.88 - −0.63) −1.61 - −0.64	6.17^ab^ (4.77–6.90) 4.77–6.90		4.50^ab^ (4.00–6.00) 4.00–6.00	−0.90^ab^ (−1.25 - −0.77) −1.25 - −0.77
* p*-value	*p* <.001*			*p* <.001*			
Mandibular Alveolar Height (Cast) (mm)							
- Median (IQR) - 95% CI of the median	5.06^ab^ (4.23–6.37) 4.30–6.33	4.23^ab^ (4.00–5.58.00.58) 4.00–5.50.00.50	−0.85^ab^ (−1.17 - −0.15) −1.09 - −0.30	4.46^ab^ (4.02–5.40) 4.02–5.40		3.82^ab^ (2.28–4.26) 2.28–4.26	−0.85^ab^ (−1.80 - −0.31) −1.80 - −0.31
* p*-value	*p* =.002*			*p* =.001*			
Overbite (cast) (mm)							
- Median (IQR) - 95% CI of the median	−4.50^ab^ (−7.94 - −3.15) −7.00 - −3.00	−1.90^ab^ (−3.81–0.50) −3.62−0.00	2.93^ab^ (1.16–5.00.16.00) 1.300–5.000.300.000	−4.00^ab^ (−5.00 - −2.00) −4.24 - −2.00		0.03^ab^ (−0.90–0.78) −0.90–0.78	3.67^ab^ (2.00–5.00) 2.000–5.000
* p*-value	*p* <.001*			*p* =.001*			
Overjet (cast) (mm)							
- Median (IQR) - 95% CI of the median	5.18^ab^ (4.34–6.01) 4.68–6.00.68.00	5.65^ab^ (3.60–6.07) 4.20–6.02	0.07^ab^ (−0.52−0.62) −0.03 −0.58	3.83^ab^ (2.40–5.76) 2.40–5.76		3.20^ab^ (2.27–5.00.27.00) 2.27–5.00.27.00	−0.61^ab^ (−1.00 - −0.13) −1.00 - −0.13
* p*-value	*p* =.778 NS			*p* =.063 NS			

*n* Number of patients, *IQR *Interquartile Range (25th percentile – 75th percentile), *p*: Probability of error (Wilcoxon Signed Ranks Test)

Superscript letters (a,b): Comparison of the two studied groups using the Mann-Whitney U test; *similar superscript at T1, T2, or change denote non statistically significant difference *

*MW* Mann-Whitney U, *WSR *Wilcoxon Signed Ranks Test

*Statistically significant (*p*<.05), *NS* Statistically significant (*p*≥.05)

*Negative values indicate a reduction in alveolar height measured on digital casts, reflecting dentoalveolar intrusion rather than extrusion*

Both groups experienced a significant reduction in upper and lower arch lengths and arch perimeters. There were no statistically noteworthy intergroup differences in these changes. The transverse arch dimension remained stable in the TS/LTPA group, while the TS group slightly increased. Additionally, both groups demonstrated a significant increase in mandibular intermolar width without significant intergroup differences. Maxillary alveolar height decreased significantly in both groups, with median changes of − 0.92 mm (IQR: − 1.83 to − 0.63 mm) in the TS/LTPA group and − 0.91 mm (IQR: − 1.25 to − 0.77 mm) in the TS group. Mandibular alveolar height demonstrated a statistically significant decrease in both groups, with a median change of − 0.85 mm (IQR: − 1.17 to − 0.15 mm) in the TS/LTPA group and − 0.85 mm (IQR: − 1.80 to − 0.31 mm) in the TS group. However, the intergroup difference was not statistically significant.

A small number of bonded spurs became unintentionally detached during the evaluation period, with failure rates recorded at 2.5% and 5.3%, respectively. In accordance with pre-treatment instructions, the dislodged spurs were expelled and promptly re-bonded. Although there is a potential risk of aspirating or swallowing detached spurs, no such incidents occurred among the participants in this study. Nevertheless, it is essential to inform patients of this possibility both before and during treatment. Aside from some plaque accumulation around the spurs, no significant adverse effects were observed. Patients were advised to maintain proper oral hygiene throughout the treatment period.

## Discussion

Multiple protocols were evaluated for the treatment of AOB in mixed dentition [32, 34, 39, 40]; nevertheless, the efficacy of banded or bonded tongue spurs and novel combined treatments is supported by limited evidence. Fixed bonded tongue spurs have been evaluated for treating AOB independently and in conjunction with bite blocks and chin cup appliances [18]. To date, no study has assessed the combined effects of a bonded tongue spur appliance with a LTPA compared to tongue spurs alone, despite the LTPA’s established role in maxillary molar eruption control and its potential to induce counterclockwise mandibular rotation [41, 42]. 

Consequently, this study investigated a novel protocol targeting a synergistic effect of vertical molar control and a mechanism to restrict anterior tongue posture. Xu et al. [42] reported a mean maxillary molar intrusion of approximately 1.5 mm, suggesting that tongue-mediated forces may contribute to posterior vertical control and anchorage reinforcement. These findings provide a biologically possible explanation for the improved maxillary molar control observed with the addition of a low transpalatal arch in the present study. However, the magnitude of such effects may be insufficient to induce clinically meaningful skeletal changes over short observation periods.

In this study, prefabricated bonded spurs were employed due to their effectiveness in modifying the anterior resting posture of the tongue, thereby preventing its interposition between the incisors. These appliances offer several clinical advantages: they are straightforward to place and remove, eliminate the need for banding and laboratory procedures, cost-effective, and can be used in both arches without compromising esthetics. Additionally, they are compatible with fixed orthodontic appliances, enhancing their versatility in interceptive and corrective treatment protocols [43].

Low TPAs receive consistent and measurable contact from the tongue, reinforcing their role as a tactile stimulus or proprioceptive cue for maintaining appropriate tongue posture. This sensory interaction forms part of a feedback loop, particularly during swallowing, whereby the tongue exerts pressure against the TPA, eliciting continuous proprioceptive input. This afferent sensory feedback facilitates spatial awareness of the tongue’s position relative to the palatal arch, thereby promoting neuromuscular re-education and reinforcing correct tongue posture over time [44].

Participants in the present study were aged between 7 and 11 years, a period corresponding to the transitional phase of mixed dentition, during which craniofacial growth remains relatively stable. This developmental stage permits the safe use of fixed or removable orthodontic appliances without adversely affecting natural growth patterns [39]. Moreover, early intervention for AOB is considered critical at this age, as reliance solely on cessation of the etiological habit is no longer sufficient to guarantee spontaneous correction, despite the possibility of self-resolution in certain cases [45].

Treatment outcomes were evaluated via digital model analysis and lateral cephalometric analysis. Maxillary and mandibular stone models were scanned using a Sirona extraoral scanner and analyzed with OrthoAnalyzer™ (3Shape), while cephalometric evaluation was conducted using WebCeph™, known for its high consistency [46]. Camardella [47] reported minimal differences between digital and plaster model measurements. Two superimposition techniques have shown high repeatability [48]. Posterior molar superimposition reflects anterior dental changes relative to posterior stability, while palatal superimposition reflects overall anterior and posterior changes relative to the palate. The latter was used herein to assess the vertical molar changes. OrthoAnalyzer has demonstrated diagnostic precision comparable to manual methods [49]. Palatal rugae were used for maxillary registration, consistent with established reliability in longitudinal digital assessments [50, 51]. The mucogingival Junction (MGJ) was employed and validated for mandibular models by Ioshida et al. [52] as a stable reference. Adel et al. [38] reported adequate intra- and inter-examiner reliability and strong ICCs for all OrthoAnalyzer™ (3Shape) linear measurements.

The groups exhibited comparable cephalometric and digital cast measurements prior to treatment to mitigate baseline bias, potentially influencing study outcomes. At the six-month follow-up period, complete AOB closure was not attained in most subjects; however, both treatment modalities elicited favorable therapeutic responses. In the TS/LTPA group, 6 out of 16 patients (37.5%) achieved complete closure of the AOB, whereas 10 patients (62.5%) exhibited notable improvement with residual open bite. In the TS group, complete closure was observed in 8 out of 14 patients (57.1%), while the remaining 6 patients (42.9%) demonstrated partial improvement without full correction. These findings indicate that both interventions were effective to varying degrees in promoting anterior bite closure.

Quantitative evaluation of overbite correction revealed a median increase of 2.93 mm for the TS/LTPA group and 3.67 mm for the TS group, as determined through digital model superimposition and analysis.

Consistently, cephalometric assessment demonstrated a median overbite improvement of 3.35 mm in the TS/LTPA group. The TS group achieved a slightly greater median increase of 3.90 mm, indicating a comparable treatment response across both treatment modalities. The very small effect size for overbite correction (Hedges g = − 0.18) supports the conclusion that both interventions produced equivalent clinical outcomes. The incomplete closure of the AOB may be attributed to the persistence of deleterious oral habits, the relatively short observation period, and the underlying divergent growth pattern and orofacial muscular imbalance observed in these patients. This evaluation utilizes the commonly adopted method for measuring overbite, which involves calculating the perpendicular distance between the incisal edges of the maxillary and mandibular incisors relative to the occlusal plane, ensuring greater accuracy [53, 54]. Pisani and Torres [5, 14] observed that bonded spurs improve overbite closure by 3.07 mm to 4.26 mm when used alone, and by 4.52 mm to 5.23 mm with a chin cup. Aliaga-Del Castillo (2021) [18] observed that the experimental and control groups had an increase of 4.84 mm in overbite. Despite implementing posterior build-ups, the overbite closure hasn’t intensified. McRae [55] assessed the efficacy of a bonded lingual spur in rectifying lingual malposition, mitigating non-nutritive sucking habits, and closing the AOB. He assessed twelve patients exhibiting non-nutritive sucking behaviors and/or abnormal tongue projection that underwent treatment with Bonded Lingual Spurs for 6 months. Improvement in overbite was noted in eleven out of twelve. Patients in the study had an average reduction of 1.38 mm in AOB over 6 months. In another study by Leite et al. (2016) [40], it was shown that the application of the spur decreased the overbite in twelve out of thirteen patients, with an average reduction of 2.14 mm over the initial 6 months: Thus, it is noteworthy that therapy with spurs seems to predict patient success after 6 months; hence, the patient response to this interceptive treatment technique determines our trial duration period (six months) based on these previous finding [32, 39, 40].

The interpretation of vertical dentoalveolar changes in this study consider the physiologic eruption of first premolars during the mixed dentition, which contributes to posterior vertical development independent of treatment mechanics. Recent evidence indicates that eruptive dental changes during growth can partially mask or counterbalance appliance-related molar vertical control, particularly in short-term interventions of ≤ 6 months. Consequently, the limited differences observed in mandibular rotation and posterior vertical parameters between groups may reflect the combined influence of growth-related eruption and treatment effects, rather than appliance inefficacy. This finding is consistent with contemporary mixed-dentition open bite studies reporting minimum vertical skeletal responses over short observation periods. Therefore, vertical treatment outcomes in early AOB interception should be interpreted cautiously, with recognition of ongoing dental eruption and growth dynamics [48, 56]. 

A minor increase of 0.62 mm in the clinical crown height of the incisors was noted, with no statistically evident differences between the groups. During the extrusive movement of the incisors, the surrounding periodontal tissues (gingiva) may not move in harmony with the teeth, potentially leading to an increase in the visible clinical crown length. This alteration is anticipated, as patients with mixed dentition frequently demonstrate underdevelopment of the vertical positioning of the maxillary incisors before the closure of the AOB [57].

In cephalometric analysis, both groups showed significant reductions in U1/FH angle (TS/LTPA: −1.00°; TS: −2.00°) and U1**-**NA (TS/LTPA: −0.50 mm; TS: −0.25 mm), indicating effective maxillary incisor uprighting. Likewise, IMPA (TS/LTPA: −4.00°; TS: −1.00°) and L1**-**NB (TS/LTPA: −0.50 mm; TS: −0.30 mm) demonstrated significant reductions, reflecting effective mandibular incisor uprighting, similar to with the results of Cassis et al. (2012) [30], but our findings oppose those of Leite et al.(2016) [40] who found no lingual tilting of maxillary incisors when using spurs; this could be linked to the different study sample’s appliance design. In the vertical plane, both groups exhibited maxillary dentoalveolar development (U1**-**PP), with median changes of 0.80 mm in TS/LTPA group and 0.95 mm in TS group, showing no statistically notable variation between groups. For mandibular dentoalveolar development (L1**-**MP), the median change was 0.95 mm in both groups; these results aligned with Aliaga-Delo Castillo et al. (2021) [18] findings.

Cephalometric and model analyses revealed differing patterns of overjet change between the two treatment groups. Cephalometrically, the TS group demonstrated a statistically significant median reduction of −1.00 mm, likely attributable to a combination of factors, including the elimination of tongue interposition, interruption of deleterious oral habits, ongoing facial maturation, and the reestablishment of orofacial muscular equilibrium [5]. Additionally, the palatal inclination of the maxillary incisors relative to the mandibular incisors may have further contributed to the observed reduction in overjet [16]. Consistently, model analysis in the TS group confirmed a greater reduction, with a median change of − 0.61 mm (IQR: − 1.00 to − 0.13 mm). In contrast, the TS/LTPA group exhibited minimal change on both assessments. Cast measurements in this Group showed a median change of 0.07 mm (IQR: − 0.52 to 0.62 mm). However, intergroup comparisons of these outcomes did not reach statistical significance. These findings align with those of Canuto et al. (2016) [16], who reported a significantly greater overjet reduction in patients treated with bonded lingual spurs compared to untreated controls. Conversely, they differ from the results of Bublitz et al. (2023) [19], who observed a non-significant decrease in overjet (–0.27 mm) in the lingual spurs group compared to a slight increase (0.12 mm) in the chin cup group (*p* =.200).

The slight overjet increase observed in the TS/LTPA group parallels outcomes seen in studies involving fixed palatal cribs used in conjunction with chin cup therapy [5]. One plausible explanation for this observation is the use of a LTPA, which may confine the tongue to a more posterior position. Consequently, the lingual pressure normally exerted by the tongue during functional activities, particularly swallowing, is diminished. This imbalance allows the perioral musculature to exert unopposed pressure on the mandibular incisors, potentially contributing to their retroclination due to the disruption in muscular equilibrium.

In cast analysis, both groups demonstrated analogous diminutions in maxillary (− 1.22 mm, − 1.04 mm) and mandibular (− 1.73 mm, − 1.60 mm) arch lengths, indicative of anterior dental uprighting. Concomitantly, a statistically non-significant reduction in arch perimeter was observed in the maxillary and mandibular dental arches, with no significant difference between the two groups. These morphological adaptations can be primarily attributed to the closure of anterior interdental spacing consequent to incisor retroclination. Furthermore, the diminution of leeway space secondary to the exfoliation of primary molars and canines, inherently more pronounced in the mandibular arch, may have further potentiated the observed dimensional reductions [22]. Vertical dentoalveolar developmental changes of 0.92 mm in the maxilla for the TS/LTPA group and 0.90 mm for the TS group, with a uniform 0.85 mm vertical development observed in the mandibular arch across both groups. The method of measurements was followed as reported by Fouda et al. (2022) [22].

True molar intrusion may alone be quantified by the vertical displacement of the molar into its alveolar bone; hence, the trifurcation point was selected as the most stable site near the center of resistance. Cusps or apices may inaccurately indicate intrusion, as they cannot differentiate between intrusion and tilting the cusp. edges or root apices [58]. The palatal plane of the maxilla and the mandibular plane, representing the essential bony framework of the jaws, are utilized to evaluate molar intrusion. In patients with untreated open bite of comparable age and follow-up duration, maxillary molar extrusion typically ranges from + 0.64 to 0.90 mm, while mandibular molar extrusion varies between + 0.44 and 0.54 mm [16, 30, 59]. Our study demonstrated significant maxillary molar control U6-pp, with a median change of + 0.40 mm in the TS/LTPA group, whereas the TS group displayed greater molar extrusion of + 0.90 mm, highlighting a significant difference between the two groups. The significant elevation in the L6-MP vertical measurement in the TS/LTPA group resulted from compensation for the controlled upper maxillary molar extrusion with a median change of + 0.50 mm in the TS/LTPA group, whereas the TS group displayed less extrusion of + 0.15 mm.

The biomechanical interpretation of the enhanced molar control achieved with the low transpalatal arch (LTPA) in this study can be explained by two primary mechanisms. First, the incorporation of an acrylic palatal pad provides an anchorage platform that increases the effective interaction with the tongue during swallowing and at rest. Previous investigations have demonstrated that integrating an acrylic pad into a transpalatal arch allows the tongue to exert consistent and measurable forces on the appliance—reaching approximately 540 g—which may contribute to up to 1.5 mm of maxillary molar intrusion during treatment [42]. This controlled vertical modulation reduces the tendency for molar extrusion, thereby limiting clockwise mandibular rotation and supporting anterior bite closure. Second, the vertical distance between the acrylic pad and the palatal vault plays a critical role in modulating force transmission. As demonstrated by Nayak et al. (2022) [60], increasing this vertical separation amplifies the moment arm and consequently enhances the vertical component of the force applied to the maxillary molars. In the present study, the acrylic pad was positioned 8 mm from the palatal mucosa, a dimension reported to meaningfully improve vertical control. This positioning likely contributed to the effective maintenance of molar vertical height and the improved anterior open-bite correction observed in the LTPA group.

These changes corresponded with a comparatively lesser increase in the y-axis, SNB, LAFH, (FH-N)-Pg measurements compared to the TS group, with an insignificant difference between both groups. These results are consistent with those of Aliaga-Del Castillo (2021) [18] and Rossato et al. (2018) [61].

Although the TS/LTPA group demonstrated improved vertical control of the maxillary molars, the control group exhibited a more favorable effective horizontal advancement of the pogonion. This intergroup difference, which favored the control group, is unexpected if the TPA promotes anterior-posterior mandibular growth through vertical growth inhibition. However, further analysis of effective vertical condylar growth revealed that the control group experienced more favorable condylar development than the TS/LTPA group. This difference suggests that enhanced vertical condylar growth in the control group may have contributed to greater mandibular rotation. Consequently, the increased horizontal displacement of pogonion seen in the control group could be due to this mandibular rotational effect driven by increased condylar growth.

The findings of the current study revealed that the TS exhibited a statistically evident reduction in the mandibular plane angle, while the TS/LTPA demonstrated an even greater decrease. However, the intergroup differences were not statistically notable. These findings align with another study indicating that a significant reduction in mandibular plane angles can be anticipated following the use of bonded spurs, regardless of posterior build-ups [18]. This phenomenon has been documented in additional studies utilizing bonded spurs [16, 61]. In contrast, only one study demonstrated a significant increase in the mandibular plane angle following bonded spur therapy. This may be linked to the distinct characteristics of their study sample.

Vertical ramus elongation measured + 0.75 mm in the TS/LTPA group, accompanied by a + 1.0° increase in gonial angle, whereas the TS group demonstrated greater elongation + 1.05 mm, with a corresponding − 1.5° decrease in gonial angulation. These outcomes are consistent with craniofacial biomechanical models, which suggest that limited vertical ramus development promotes clockwise mandibular rotation, thereby increasing gonial angle obtuseness [62]. The inverse correlation between ramus growth magnitude and gonial angle alteration requires confirmation via prospective controlled trials to mitigate bias and validate clinical significance.

Dentoalveolar measurements showed that the improvement in overbite observed in both the experimental and control groups was associated with palatal tipping and extrusion of the maxillary incisors, along with lingual inclination and extrusion, aligning with previous findings on the effects of these treatment approaches [3, 16, 30, 40, 59]. Our Findings indicated that overbite correction was predominantly attained through dentoalveolar effects. This suggests that early open bite correction is more manageable, as it is typically predominantly dental, and the increased growth potential can facilitate the correction. Clinicians advocate for spur therapy to modify tongue behavior, rectify the open bite, and enhance treatment stability [63–66]. Nonetheless, some individuals remain apprehensive regarding the responses of patients and parents to this orthodontic device [67].

The comparative acceptance evaluation between the two groups demonstrated significantly higher patient satisfaction in the TS group concerning noticeability, speech, eating, and pain experience, and gum bleeding compared to the TS/LTPA Group [37], with discomfort lasting temporarily for up to 7 days in most patients [68].

The results suggest that combining a LTPA with bonded tongue spurs provided better vertical molar control than the TS groups. However, the difference between the two groups did not differ in a statistically meaningful way, possibly as a result of compensatory eruption of the mandibular molars. Clinically, bonded spurs are generally more convenient, as they eliminate the need for banding procedures and laboratory fabrication.

### Limitations

This study has specific methodological constraints that should be acknowledged. First, the absence of a non-intervention control group restricts the ability to separate treatment effects from growth-related changes; an untreated arm was not feasible for ethical reasons. Second, the six-month follow-up may not capture the full course of open-bite correction or its long-term stability. Third, the presence of residual open bite in some participants indicates that a longer observation period may be necessary to evaluate the complete treatment response, particularly in patients with persistent functional habits or a vertical growth pattern. Studies with extended follow-up intervals and broader samples are needed to validate and expand these findings.

### Clinical significance

The findings of this trial indicate that bonded tongue spurs alone produced therapeutic changes comparable to the combined TS/LTPA approach, while being associated with fewer functional disturbances and higher patient acceptance. The addition of a low transpalatal arch offers increased control of maxillary molar vertical position, but this advantage should be balanced against the increased discomfort and reduced adaptability reported with the combined appliance. Within the mixed-dentition stage, bonded tongue spurs can be considered an efficient interceptive option for anterior open-bite management when patient comfort and compliance are prioritized, whereas the TS/LTPA combination may be reserved for cases where enhanced vertical molar control is clinically required.

## Conclusion

Both the combination of low TPA with bonded TS and the use of bonded tongue spurs alone demonstrated comparable effectiveness in improving overbite during the early interceptive treatment of AOB. The combination of bonded tongue spurs with low TPA elicited a statistically significant improvement in vertical maxillary molar control; however, it did not result in a clinically meaningful counterclockwise mandibular rotation due to compensatory lower molar eruption when compared to tongue spurs alone over the 6-month observation period. Bonded tongue spurs alone effectively manage developing anterior open bite associated with abnormal tongue posture and minimal posterior vertical excess, offering comparable outcomes to combined approaches with simpler design and higher patient acceptance. Low transpalatal arches should be reserved for cases requiring posterior vertical control, such as increased maxillary molar eruption or vertical growth tendency.

### Limitations and generalizability

The follow-up period was short, preventing long-term assessment of treatment stability and skeletal changes. This study focused primarily on dentoalveolar effects; therefore, conclusions regarding skeletal growth cannot be drawn. Future studies with extended follow-up are warranted to evaluate skeletal effects comprehensively. n: Number of patients IQR: Interquartile Range (25th percentile – 75th percentile). *Statistically significant (*p* <.05). NS: Statistically significant (*p* ≥.05).

## Supplementary Information

Supplementary Material 1.

## Data Availability

The de-identified individual participant data, along with the data dictionary, statistical code, and other related materials, will be available upon reasonable request. Interested researchers can contact the corresponding author. Access will be granted following approval of a methodologically sound proposal and signing of a data sharing agreement. Data will be shared through a secure institutional repository or encrypted file transfer.

## Abbreviations

TS/LTPA: Tongue spurs and low trans palatal arch
TS: Tongue spurs
AOB: Anterior open bite
VHA: Vertical holding appliance
Y-AXIS: Y-Axis of Growth
LAFH: Lower Anterior Facial Height
BLS: Bonded Lingual Spurs
U6-PP: Maxillary First Molar to Palatal Plane
L6-MP: Mandibular First molar to mandibular plane
(FH- N)-Pg: The perpendicular linear distance from Pogonion (Pg) to a line dropped perpendicularly from Nasion (N) onto the Frankfort Horizontal (FH) plane

## References

[1] Subtelny JD, Sakuda M. Open-bite: diagnosis and treatment. Am J Orthod. 1964;50(5):337–58.

[2] Lone IM, Zohud O, Midlej K, Paddenberg E, Krohn S, Kirschneck C, et al. Anterior open bite malocclusion: from clinical treatment strategies towards the dissection of the genetic bases of the disease using human and collaborative cross mice cohorts. J Pers Med. 2023;13(11):1617. 38003932 10.3390/jpm13111617 PMC10672619

[3] Tausche E, Luck O, Harzer W. Prevalence of malocclusions in the early mixed dentition and orthodontic treatment need. Eur J Orthod. 2004;26(3):237–44. 15222706 10.1093/ejo/26.3.237

[4] Lorente AA, Cortes O, Guzmán S, Vicente A, Garrido N. Oral malocclusion and its relation to nutritive and non-nutritive habits in school children. Open J Dent Oral Med. 2019;7(1):1–8.

[5] Torres FC, Almeida RR, Almeida-Pedrin RR, Pedrin F, Paranhos LR. Dentoalveolar comparative study between removable and fixed cribs, associated to chincup, in anterior open bite treatment. J Appl Oral Sci. 2012;20(5):531–7. 23138739 10.1590/S1678-77572012000500007 PMC3881795

[6] Lin L, Zhao T, Qin D, Hua F, He H. The impact of mouth breathing on dentofacial development: a concise review. Front Public Health. 2022;10:929165. 36159237 10.3389/fpubh.2022.929165 PMC9498581

[7] Moss ML. The functional matrix hypothesis revisited. 1. The role of mechanotransduction. Am J Orthod Dentofacial Orthop. 1997;112(1):8–11. 9228835 10.1016/s0889-5406(97)70267-1

[8] Karanam LS Venugopalans. Absence of anterior guidance and its effects on early discal changes in temporomandibular joint: A Cross-sectional study. J Clin Diagn Res. 2025;19(6).

[9] Cozza P, Baccetti T, Franchi L, Mucedero M, Polimeni A. Sucking habits and facial hyperdivergency as risk factors for anterior open bite in the mixed dentition. Am J Orthod Dentofacial Orthop. 2005;128(4):517–9. 16214636 10.1016/j.ajodo.2005.04.032

[10] Wang H, Qiao X, Qi S, Zhang X, Li S. Effect of adenoid hypertrophy on the upper airway and craniomaxillofacial region. Transl Pediatr. 2021;10(10):2563–72. 34765480 10.21037/tp-21-437 PMC8578754

[11] Valério P, Peričić TP, Rossi A, Grippaudo C, dS T J, do Nascimento IJB. The effectiveness of early intervention on malocclusion and its impact on craniofacial growth: a systematic review. Contemp Pediatr Dent. 2021;2021:1–18.

[12] Lentini-Oliveira DA, Carvalho FR, Rodrigues CG, Ye Q, Prado LB, Prado GF, et al. Orthodontic and orthopaedic treatment for anterior open bite in children. Cochrane Database Syst Rev. 2014;2014(9):CD005515. 25247473 10.1002/14651858.CD005515.pub3 PMC10964129

[13] Feres MF, Abreu LG, Insabralde NM, Almeida MR, Flores-Mir C. Effectiveness of the open bite treatment in growing children and adolescents. A systematic review. Eur J Orthod. 2016;38(3):237–50. 26136439 10.1093/ejo/cjv048 PMC4914905

[14] Pisani L, Bonaccorso L, Fastuca R, Spena R, Lombardo L, Caprioglio A. Systematic review for orthodontic and orthopedic treatments for anterior open bite in the mixed dentition. Prog Orthod. 2016;17(1):28. 27615261 10.1186/s40510-016-0142-0 PMC5027197

[15] Feres MF, Abreu LG, Insabralde NM, de Almeida MR, Flores-Mir C. Effectiveness of open bite correction when managing deleterious oral habits in growing children and adolescents: a systematic review and meta-analysis. Eur J Orthod. 2017;39(1):31–42. 26846264 10.1093/ejo/cjw005

[16] Canuto LF, Janson G, de Lima NS, de Almeida RR, Cancado RH. Anterior open-bite treatment with bonded vs conventional lingual spurs: a comparative study. Am J Orthod Dentofacial Orthop. 2016;149(6):847–55. 27241995 10.1016/j.ajodo.2015.11.026

[17] Artese A, Drummond S, Nascimento JM, Artese F. Criteria for diagnosing and treating anterior open bite with stability. Dent Press J Orthod. 2011;16:136–61.

[18] Aliaga-Del Castillo A, Vilanova L, Miranda F, Arriola-Guillen LE, Garib D, Janson G. Dentoskeletal changes in open bite treatment using spurs and posterior build-ups: a randomized clinical trial. Am J Orthod Dentofacial Orthop. 2021;159(1):10–20. 33221096 10.1016/j.ajodo.2020.06.031

[19] Bublitz TCF, Fernandes TMF, Oltramari PVP, Almeida MRd, Almeida-Pedrin RR, Ladewig VdM, et al. Anterior open-bite treatment with lingual spurs and chincup: a prospective, randomized digital model study. J Health Sci. 2023;25(1):02–9.

[20] Schulz KF, Altman DG, Moher D, Group* C. CONSORT 2010 statement: updated guidelines for reporting parallel group randomized trials. Ann Intern Med. 2010;152(11):726–32. 20335313 10.7326/0003-4819-152-11-201006010-00232

[21] Faul F, Erdfelder E, Lang AG, Buchner A. G*power 3: a flexible statistical power analysis program for the social, behavioral, and biomedical sciences. Behav Res Methods. 2007;39(2):175–91. 17695343 10.3758/bf03193146

[22] Fouda AS, Afify AK, Aboulfotouh MH, Attia KH, Abouelezz AM, Elkordy SA. Dental arch changes after anterior open bite treatment in the mixed dentition produced by miniscrew-supported palatal crib vs conventional fixed palatal crib. Angle Orthod. 2022;92(4):487–96. 35130348 10.2319/082321-659.1 PMC9235388

[23] Schulz KF, Grimes DA. Generation of allocation sequences in randomised trials: chance, not choice. Lancet. 2002;359(9305):515–9. 11853818 10.1016/S0140-6736(02)07683-3

[24] Schulz KF, Grimes DA. Allocation concealment in randomised trials: defending against deciphering. Lancet. 2002;359(9306):614–8. 11867132 10.1016/S0140-6736(02)07750-4

[25] Karanicolas PJ, Farrokhyar F, Bhandari M. Blinding: who, what, when, why, how? Can J Surg. 2010;53(5):345. 20858381 PMC2947122

[26] IBM Corp. IBM SPSS statistics for Windows, version 25.0. Armonk, NY: IBM Corp.; Released; 2017.

[27] Field A. Discovering Statistics Using IBM SPSS Statistics. 4th ed. London, California, New Delhi: SAGE Publications Ltd; 2013.

[28] Mann HB, Whitney DR. On a test of whether one of two random variables is stochastically larger than the other. Ann Math Stat. 1947. 10.1214/aoms/1177730491.

[29] Wilcoxon F. Individual comparisons by ranking methods. Biometrics Bull. 1945;1(6):80–3. 18903631

[30] Cassis MA, de Almeida RR, Janson G, de Almeida-Pedrin RR, de Almeida MR. Treatment effects of bonded spurs associated with high-pull chincup therapy in the treatment of patients with anterior open bite. Am J Orthod Dentofacial Orthop. 2012;142(4):487–93. 22999672 10.1016/j.ajodo.2012.04.022

[31] Vela-Hernandez A, Lopez-Garcia R, Garcia-Sanz V, Paredes-Gallardo V, Lasagabaster-Latorre F. Nonsurgical treatment of skeletal anterior open bite in adult patients: posterior build-ups. Angle Orthod. 2017;87(1):33–40. 27434615 10.2319/030316-188.1 PMC8388603

[32] Meyer-Marcotty P, Hartmann J, Stellzig-Eisenhauer A. Dentoalveolar open bite treatment with spur appliances. J Orofac Orthop. 2007;68(6):510–21. 18034291 10.1007/s00056-007-0707-0

[33] Haryett RD, Hansen FC, Davidson PO. Chronic thumb-sucking. A second report on treatment and its psychological effects. Am J Orthod. 1970;57(2):164–78. 5262793 10.1016/0002-9416(70)90263-0

[34] Slaviero T, Fernandes TM, Oltramari-Navarro PV, de Castro AC, Conti F, Poleti ML, et al. Dimensional changes of dental arches produced by fixed and removable palatal cribs: A prospective, randomized, controlled study. Angle Orthod. 2017;87(2):215–22. 27598906 10.2319/060116-438.1 PMC8384369

[35] Rakosi T. Bedeutung der angulären und linearen messungen in derdento-skelettalen analyse. In: Rakosi T, editor. Atlas und Anleitungzur Praktischen Fernröntgenanalyse. München: Hanser; 1988. pp. 58–98.

[36] Beckmann SH, Kuitert RB, Prahl-Andersen B, Segner D, The RP, Tuinzing DB. Alveolar and skeletal dimensions associated with lower face height. Am J Orthod Dentofacial Orthop. 1998;113(5):498–506. 9598607 10.1016/s0889-5406(98)70260-4

[37] Nasser M, Yousry TN, Ismail H. Patient acceptance and satisfaction: a comparative study of low transpalatal arch with tongue Spurs versus tongue Spurs alone in developing anterior open bite treatment. Egypt Orthod J. 2025;67(1):157–77.

[38] Adel SM, Vaid NR, El-Harouni N, Kassem H, Zaher AR. Digital model superimpositions: are different software algorithms equally accurate in quantifying linear tooth movements? BMC Oral Health. 2022;22(1):103. 35361187 10.1186/s12903-022-02129-x PMC8973572

[39] Huang GJ, Justus R, Kennedy DB, Kokich VG. Stability of anterior openbite treated with crib therapy. Angle Orthod. 1990;60(1):17–24. discussion 5–6. 2316899 10.1043/0003-3219(1990)060<0017:SOAOTW>2.0.CO;2

[40] Leite JS, Matiussi LB, Salem AC, Provenzano MG, Ramos AL. Effects of palatal crib and bonded Spurs in early treatment of anterior open bite: a prospective randomized clinical study. Angle Orthod. 2016;86(5):734–9. 26719946 10.2319/031815-170.1 PMC8600830

[41] Wise JB, Magness WB, Powers JM. Maxillary molar vertical control with the use of transpalatal arches. Am J Orthod Dentofacial Orthop. 1994;106(4):403–8. 7942656 10.1016/S0889-5406(94)70062-1

[42] Xu K, Zeng J, Xu T. Effect of an intraoral appliance on tongue pressure measured by force exerted during swallowing. Am J Orthod Dentofacial Orthop. 2016;149(1):55–61. 26718378 10.1016/j.ajodo.2015.06.023

[43] Dias FA, Assis Urnau FD, Pedron Oltramari PV, Lupion Poleti M, Rodrigues de Almeida M, Freire Fernandes TM. Stability of early treatment of anterior open bite: clinical performance of bonded lingual spurs. J Orthod. 2019;46(1):68–73. 31056074 10.1177/1465312519827601

[44] Chiba Y, Motoyoshi M, Namura S. Tongue pressure on loop of transpalatal arch during deglutition. Am J Orthod Dentofac Orthop. 2003;123(1):29–34. 10.1067/mod.2003.51 12532060

[45] Fränkel R, Fränkel C. Orofacial orthopedics with the function regulator. Karger Medical and Scientific; 1989.

[46] Mahto RK, Kafle D, Giri A, Luintel S, Karki A. Evaluation of fully automated cephalometric measurements obtained from web-based artificial intelligence driven platform. BMC Oral Health. 2022;22(1):132. 35440037 10.1186/s12903-022-02170-w PMC9020017

[47] Camardella LT, Sa M, Guimaraes LC, Vilella BS, Vilella OV. Agreement in the determination of preformed wire shape templates on plaster models and customized digital arch form diagrams on digital models. Am J Orthod Dentofac Orthop. 2018;153(3):377–86. 10.1016/j.ajodo.2017.07.019 29501113

[48] Aliaga-Del Castillo A, Janson G, Vilanova L, Cevidanes L, Yatabe M, Garib D, et al. Three-dimensional Dentoalveolar changes in open bite treatment in mixed dentition, Spurs/posterior build-ups versus Spurs alone: 1-year follow-up randomized clinical trial. Sci Rep. 2022;12(1):12378. 35858941 10.1038/s41598-022-15988-9 PMC9300740

[49] Barreto MS, Faber J, Vogel CJ, Araujo TM. Reliability of digital orthodontic setups. Angle Orthod. 2016;86(2):255–9. 26042573 10.2319/120914-890.1 PMC8603624

[50] Vasilakos G, Schilling R, Halazonetis D, Gkantidis N. Assessment of different techniques for 3D superimposition of serial digital maxillary dental casts on palatal structures. Sci Rep. 2017;7(1):5838. 28724930 10.1038/s41598-017-06013-5 PMC5517608

[51] Jang I, Tanaka M, Koga Y, Iijima S, Yozgatian JH, Cha BK, et al. A novel method for the assessment of three-dimensional tooth movement during orthodontic treatment. Angle Orthod. 2009;79(3):447–53. 19413387 10.2319/042308-225.1

[52] Ioshida M, Munoz BA, Rios H, Cevidanes L, Aristizabal JF, Rey D, et al. Accuracy and reliability of mandibular digital model registration with use of the mucogingival junction as the reference. Oral Surg Oral Med Oral Pathol Oral Radiol. 2019;127(4):351–60. 30472195 10.1016/j.oooo.2018.10.003

[53] Kucukkeles N, Acar A, Demirkaya AA, Evrenol B, Enacar A. Cephalometric evaluation of open bite treatment with NiTi arch wires and anterior elastics. Am J Orthod Dentofac Orthop. 1999;116(5):555–62. 10.1016/s0889-5406(99)70189-7 10547517

[54] Janson G, Valarelli FP, Beltrao RT, de Freitas MR, Henriques JF. Stability of anterior open-bite extraction and nonextraction treatment in the permanent dentition. Am J Orthod Dentofacial Orthop. 2006;129(6):768–74. 16769495 10.1016/j.ajodo.2004.11.031

[55] McRae EJ. Bondable lingual spur therapy to treat anterior open bite. Marquette University; 2010.

[56] Yousif ASF, Hassan AFF, Idress LTMA, Elhassan IK, Elnour R, Omer EAMS et al. Early orthodontic and orthopedic interventions for anterior open bite in the mixed dentition: A systematic review. Cureus. 2025;17(11). 10.7759/cureus.96806 PMC12701947 41399602

[57] de Brito Vasconcelos J, de Almeida-Pedrin RR, Poleti T, Oltramari P, de Castro Conti ACF, Bicheline MHB, et al. A prospective clinical trial of the effects produced by the extrusion arch in the treatment of anterior open bite. Prog Orthod. 2020;21(1):39. 33078213 10.1186/s40510-020-00339-z PMC7573098

[58] Burstone CR. Deep overbite correction by intrusion. Am J Orthod. 1977;72(1):1–22. 267433 10.1016/0002-9416(77)90121-x

[59] Insabralde NM, de Almeida RR, Henriques JF, Fernandes TM, Flores-Mir C, de Almeida MR. Dentoskeletal effects produced by removable palatal crib, bonded spurs, and chincup therapy in growing children with anterior open bite. Angle Orthod. 2016;86(6):969–75. 27159552 10.2319/011916-49.1 PMC8597351

[60] Nayak TK, Nanda SB, Sinha A, Pradhan R, Pattanaik S, Sahoo SN. Comparative evaluation of transpalatal arch and vertical holding appliance at different heights. J Taibah Univ Med Sci. 2022;17(3):401–7. 35722232 10.1016/j.jtumed.2021.11.004 PMC9170769

[61] Rossato PH, Fernandes TMF, Urnau FDA, de Castro AC, Conti F, de Almeida RR, et al. Dentoalveolar effects produced by different appliances on early treatment of anterior open bite: a randomized clinical trial. Angle Orthod. 2018;88(6):684–91. 29911909 10.2319/101317-691.1 PMC8174065

[62] Bjork A, Skieller V. Normal and abnormal growth of the mandible. A synthesis of longitudinal cephalometric implant studies over a period of 25 years. Eur J Orthod. 1983;5(1):1–46. 6572593 10.1093/ejo/5.1.1

[63] Justus R. Correction of anterior open bite with spurs: long-term stability. World J Orthod. 2001;2(3):219–31.

[64] Sayin MO, Akin E, Karacay S, Bulakbasi N. Initial effects of the tongue crib on tongue movements during deglutition: a cine-magnetic resonance imaging study. Angle Orthod. 2006;76(3):400–5. 16637718 10.1043/0003-3219(2006)076[0400:IEOTTC]2.0.CO;2

[65] Haryett RD, Hansen FC, Davidson PO, Sandilands ML. Chronic thumb-sucking: the psychologic effects and the relative effectiveness of various methods of treatment. Am J Orthod. 1967;53(8):569–85. 4951439 10.1016/0002-9416(67)90069-3

[66] Alawy SB, El-Desouky SS, Kabbash IA, Hadwa SM. Effects of tongue tamers and customized bonded spurs as an early treatment of anterior open bite: a randomized clinical study. BMC Oral Health. 2025;25(1):76. 39819458 10.1186/s12903-024-05389-x PMC11737204

[67] Araujo EA, Andrade I Jr., Brito Gde M, Guerra L, Horta MC. Perception of discomfort during orthodontic treatment with tongue spurs. Orthodontics (Chic). 2011;12(3):260–7. 22022697

[68] Brown DF, Moerenhout RG. The pain experience and psychological adjustment to orthodontic treatment of preadolescents, adolescents, and adults. Am J Orthod Dentofacial Orthop. 1991;100(4):349–56. 1927986 10.1016/0889-5406(91)70073-6

